# Lichen Extracts Containing Volatile Compounds Induce Oxidative Stress and Modulate the Growth of *Microcystis aeruginosa* and *Chlorella sorokiniana*

**DOI:** 10.3390/ijms27114790

**Published:** 2026-05-26

**Authors:** Yasser Essadki, El Mehdi Darrag, Soukaina El Amrani Zerrifi, Mohamed Haida, Aafaf Krimech, Rosario Martins, Alexandre Campos, Vitor Vasconcelos, Noureddine Bouaïcha, Abdelaziz Baçaoui, Abdelilah Meddich, Brahim Oudra, Zakaria Tazart, Fatima El Khalloufi

**Affiliations:** 1 Water Sciences, Microbial Biotechnologies and Sustainability of Natural Resources Laboratory (Aquabiotech), Faculty of Sciences Semlalia of Marrakech, Cadi Ayyad University, Av. Prince My Abdellah, P.O. Box 2390, Marrakech 40000, Morocco; yasser.essadki@ced.uca.ma (Y.E.); s.elamrani@sante.gov.ma (S.E.A.Z.); mohammed.haida@ced.uca.ma (M.H.); aafaf.krimech@edu.uca.ac.ma (A.K.); oudra@uca.ac.ma (B.O.); 2 Laboratory of Applied Chemistry and Biomass, Faculty of Sciences Semlalia Marrakech, Cadi Ayyad University, Bd Prince Moulay Abdellah, Marrakech 40000, Morocco; elmehdi.darrag@ced.uca.ma (E.M.D.); bacaoui@uca.ma (A.B.); 3 Abiotic Stress Physiology Team, Laboratory of Excellence in Agrobiotechnology and Bioengineering, AgroBiotech Center, CNRST-Labeled Research Unit (URL05-CNRST), Faculty of Sciences Semlalia of Marrakech, Cadi Ayyad University, Bd Prince Moulay Abdellah, Marrakech 40000, Morocco; a.meddich@uca.ma; 4 Higher Institute of Nurses Professions and Health Techniques of Dakhla, Av. Al walaa, Dakhla 73000, Morocco; 5 National Center for Studies and Research on Water and Energy, Cadi Ayyad University, Avenue Abdelkrim Elkhatabi, P.O. Box 511, Marrakech 40000, Morocco; 6 Interdisciplinary Centre of Marine and Environmental Research (CIIMAR/CIMAR), University of Porto, Terminal de Cruzeiros do Porto de Leixões, 4450-208 Matosinhos, Portugal; mrm@ess.ipp.pt (R.M.); acampos@ciimar.up.pt (A.C.); vmvascon@fc.up.pt (V.V.); 7 ESS, Escola Superior de Saúde, Polytechnic Institute of Porto, R. Dr. António Bernardino de Almeida 400, 4200-072 Porto, Portugal; 8 Department of Biology, Faculty of Sciences, University of Porto, Rua Campo Alegre s/n, 4169-007 Porto, Portugal; 9 Laboratoire Écologie, Société et Évolution, UMR 8079, Université Paris-Saclay, CNRS, AgroParisTech, 91190 Gif-sur-Yvette, France; noureddine.bouaicha@universite-paris-saclay.fr; 10 Plant Stress Physiology Laboratory, AgroBioSciences, Mohammed VI Polytechnic University, Benguerir 43150, Morocco; 11 Natural Resources Engineering and Environmental Impacts Team, Multidisciplinary Research and Innovation Laboratory, Polydisciplinary Faculty of Khouribga, Sultan Moulay Slimane University of Beni Mellal, Bd 2 Mars, Khouribga 25000, Morocco; f.elkhalloufi@usms.ma

**Keywords:** *Pseudevernia furfuracea*, volatile organic compounds (VOCs), anti-cyanobacterial activity, anti-microalgae, oxidative stress, algal proliferation, molecular docking

## Abstract

This study evaluates volatile extracts (HE1 and HE2) from the lichen *Pseudevernia furfuracea* as eco-friendly agents to control algal proliferation, specifically targeting the cyanobacterium *Microcystis aeruginosa* and the green microalga *Chlorella sorokiniana*. Both extracts exhibited potent anti-microalgal activity against the two species with a minimum inhibitory concentration (MIC) ranging from 375 to 750 µg/mL. Furthermore, both extracts reduced cell density by more than 98% after eight days of treatment. Chlorophyll *a* and protein levels decreased significantly (>80%) in both species, indicating suppression of pigment synthesis. However, their physiological responses were distinct: *M. aeruginosa* underwent early acute oxidative stress and severe membrane damage, while *C. sorokiniana* exhibited delayed oxidative activation and a negative growth rate, suggesting non-lytic metabolic inhibition. An in silico study by molecular docking of the most abundant compounds identified in these volatile extracts, such as terpenoids (abietatriene, δ-cadinene) and a phenolic compound (atraric acid), showed that these compounds interact with vital cellular targets in *M. aeruginosa* and *C. sorokiniana* and likely contribute to the effects observed in these two species. Predictive toxicity by applying the ADMET framework confirmed the favorable bioavailability and low acute toxicity of these volatile compounds. Therefore, *P. furfuracea* volatiles are promising, species-specific, and environmentally safe candidates for mitigating aquatic algal proliferation through targeted oxidative and metabolic interference.

## 1. Introduction

The proliferation of harmful algal blooms (HABs), including cyanobacterial blooms (HCBs) in marine and freshwater ecosystems, has become a critical global environmental concern, exacerbated by eutrophication and rising global temperatures [1,2,3,4]. In freshwater ecosystems, these blooms are often dominated by cyanobacteria such as *Microcystis aeruginosa* that pose severe threats to aquatic ecosystems, public health, and local economies through the production of potent toxins and the depletion of dissolved oxygen [5]. Current control strategies largely rely on synthetic algicides (e.g., copper sulfate), which, despite their efficacy, are non-selective and can accumulate in the environment, causing long-term ecological damage [6,7]. Consequently, there is an urgent need to explore environmentally friendly, biodegradable alternatives derived from natural sources.

In the search for novel bioactive compounds, lichens represented a promising yet underutilized reservoir [8]. Lichens are unique symbiotic associations capable of synthesizing over one thousand known secondary metabolites, including depsides, depsidones, dibenzofurans, and terpenes [9,10]. These compounds have attracted significant pharmacological attention for their broad spectrum of biological activities, including antibacterial, antifungal, antiviral, antioxidant, and cytotoxic properties [11,12,13]. However, despite this extensive bioactivity profile, the potential of lichen metabolites to inhibit photosynthetic microorganisms remains largely unexplored.

The rationale for investigating lichens as a source of anti-microalgal agents is rooted in their fundamental biology. Lichens are complex composite organisms formed through a stable association between a fungal partner (mycobiont) and a photosynthetic partner (photobiont) [14,15]. This association is not merely structural but is the result of a finely balanced equilibrium maintained through strict metabolic regulation [16,17,18]. Recent evidence suggests that specific lichen metabolites may regulate photobiont proliferation within the thallus, acting as chemical cues to maintain metabolic balance and potentially prevent the overgrowth of the algal partner [19,20]. If lichen metabolites have evolved to regulate symbiotic algae, it is biologically plausible that they may also possess allelopathic activity against free-living phytoplankton.

Despite this, relatively few studies have examined the effects of lichen extracts on ecologically relevant cyanobacteria or microalgae [21,22]. Furthermore, the biological role of volatile compounds from lichens in this context remains poorly understood. Volatile compounds, often lipophilic and capable of crossing cell membranes, play crucial roles in chemical communication and allelopathy in aquatic systems [23]. To our knowledge, no prior work has evaluated the algicidal or anti-cyanobacterial potential of volatile extracts from lichens.

The present study aims to evaluate volatile extracts from the lichen *Pseudevernia furfuracea* (L.) Zopf as a potential source of novel anti-cyanobacterial and anti-microalgal agents. We investigated, for the first time, the inhibitory effects of two volatile extracts (HE1 and HE2) from the lichen *P. furfuracea* harvested from two Moroccan biotopes on the growth of the cyanobacterium *Microcystis aeruginosa* and the green alga *Chlorella sorokiniana*. Beyond growth inhibition, we assessed the physiological mechanisms of action of these two extracts by measuring chlorophyll *a* and protein levels and oxidative stress biomarkers, such as superoxide dismutase and catalase activities and malondialdehyde levels. Finally, molecular docking and predictive toxicity assessment using the ADMET framework were employed to elucidate potential molecular interactions. This integrative approach provides new insights into the ecological functions of lichen volatile compounds and their prospective application as natural biocontrol agents in aquatic management.

## 2. Results

### 2.1. Chemical Profile of HE1 and HE2 Extracts

The chemical profiles of the volatile extracts HE1 and HE2 were previously characterized and are summarized in Table 1 [24]. While atraric acid and chloroatranol constitute the major compounds of the HE1 and HE2 extracts, with relative abundances ranging from 73.53 to 56.95% and from 19.80 to 24.38%, respectively, a diverse array of minor terpenoids and aromatic constituents was also identified. To provide a comprehensive understanding of the lichen’s bioactive potential, all identified compounds, regardless of their relative abundance, were subsequently evaluated through in silico molecular docking to investigate their specific roles in growth modulation and the induction of oxidative stress.

### 2.2. Qualitative Assessment of Anti-Cyanobacterial and Anti-Algal Activity Using the Disc Diffusion Method

**Figure 1:**
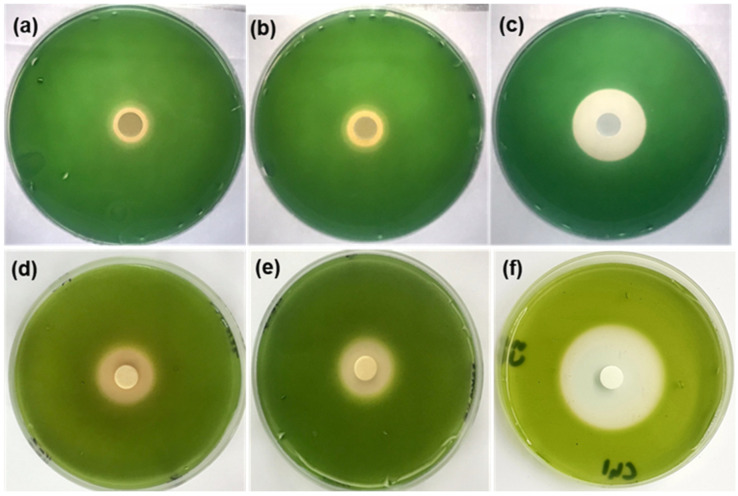
Anti-cyanobacterial (**a**–**c**) and anti-microalgal (**d**–**f**) activity against *M. aeruginosa* and *C. sorokiniana*, respectively. (**a**,**d**): HE1 (400 µg), (**b**,**e**): HE2 (400 µg), and (**c**,**f**): PC (copper sulfate) at 600 µg.

The anti-cyanobacterial (*M. aeruginosa*) and anti-microalgal (*C. sorokiniana*) activity of HE1 and HE2 extracts was determined qualitatively by the disc diffusion method. The results presented in Figure 1 and Table 2 show that both extracts (HE1 and HE2) with 20 µL at 1 mg/mL (400 µg) induced inhibitory activity against the two species *M. aeruginosa* and *C. sorokiniana*. The inhibition zone diameters (IZDs) at this dose were 12.56 ± 0.82 and 13.66 ± 0.28 mm against *M. aeruginosa* for HE1 and HE2, respectively, while against *C. sorokiniana*, the IZDs were higher, with values of 20.7 ± 0.24 and 20.9 ± 0.63 mm for HE1 and HE2, respectively. The HE2 extract exhibits a significantly larger IZD on *M. aeruginosa* compared to the HE1 extract (Table 2). However, in *C. sorokiniana*, although the IZD is larger than in Microcystis, it remains similar for both extracts. Copper sulfate at 600 µg, taken as a positive control, induces a greater IZD in both species than that induced by the two extracts, but with a significantly greater IZD in *C. sorokiniana* (Table 2).

### 2.3. Minimum Inhibitory Concentration and Minimum Microbicidal Concentration

The activity of HE1 and HE2 extracts against *M. aeruginosa* and *C. sorokiniana* in liquid medium was determined quantitatively using the broth microdilution assay. After 8 days of incubation, the minimum inhibitory concentration (MIC) was observed, and the results are summarized in Table 3. The HE1 extract revealed MICs of 375 and 750 µg/mL against *M. aeruginosa* and *C. sorokiniana*, respectively, suggesting that the two species exhibit different levels of sensitivity to the extract, while the HE2 extract showed a similar MIC (375 µg/mL) against both *M. aeruginosa* and *C. sorokiniana*. Copper sulfate had an MIC of 4.688 µg/mL. In all the treatments, the MIC was equal to the minimum microbicidal concentration (MMC), suggesting that the compounds have microbicidal potential.

### 2.4. Growth Monitoring and Biochemical Parameters

#### 2.4.1. Growth Parameters

**Figure 2:**
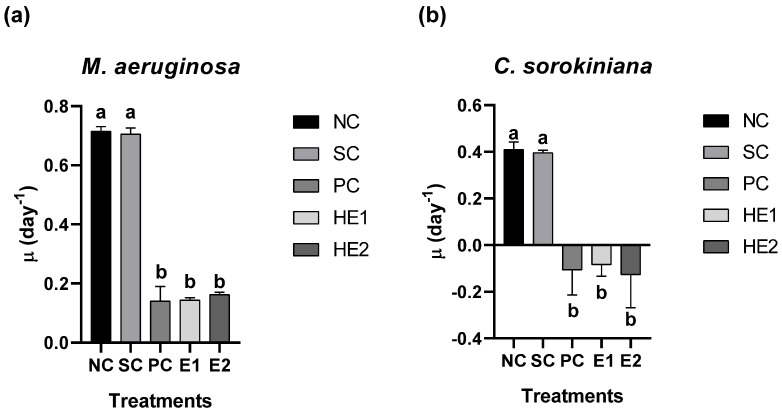
Effect of HE1 and HE2 extracts at minimum inhibitory concentration on the growth rate (µ, expressed in day^−1^) of *M. aeruginosa* (**a**) and *C. sorokiniana* (**b**) in liquid medium. Each value is the mean ± SD of three replicates. Different letters represent statistically significant differences (*p* < 0.001). NC, negative control; SC, solvent control (DMSO); PC, positive control (CuSO_4_) at minimum inhibitory concentration.

The monitoring of *M. aeruginosa* and *C. sorokiniana* growth was conducted every 48 h for 8 days. Growth rates (Figure 2) and inhibition rates (Table 4 and Table 5) were then determined in both species. For *M. aeruginosa*, the cell density in the negative control group reached a maximum of 6267.93 × 10^5^ cells/mL after 8 days, corresponding to a specific growth rate of 0.71 per day. In contrast, treatments with lichen extracts significantly suppressed growth; by day 8, cell densities reached only 65.22 × 10^5^ cells/mL for HE1 and 75.35 × 10^5^ cells/mL for HE2, with growth rates of 0.144 per day and 0.162 per day, respectively (*p* < 0.05). These values were comparable to the positive control (copper sulfate), which reached a density of 63.7 × 10^5^ cells/mL with a growth rate of 0.14 per day. The inhibition rate (IR) for HE1 was 76.93% at day 2, gradually increasing to a maximum of 98.95% by day 8. Similarly, HE2 exhibited an IR of 79.62% at day 2, reaching a maximum of 98.79% at the conclusion of the treatment.

For *C. sorokiniana*, the cell density of the negative control reached a maximum of 499.03 × 10^5^ cells/mL at day 8, with a growth rate of 0.41 per day. On the other hand, in the presence of HE1 extract, cell density reached only 9.53 × 10^5^ cells/mL at day 8, with a growth rate of −0.08 per day. Similarly, in the presence of HE2 extract, cell density reached 6.77 × 10^5^ cells/mL at day 8, with a growth rate of −0.12 per day. However, in the presence of copper sulfate (PC), the cell density of *C. sorokiniana* reached 7.97 × 10^5^ cells/mL at day 8, with a growth rate of −0.106 per day. The inhibition rate of HE1 extract on this algal species at day 2 reached a value of 88.59%, gradually increasing to a maximum of 98.09% at the last day (Table 5). The IR of HE2 extract reached 90.21% at day 2, increasing to a maximum of 98.77% at day 6, and decreasing to 98.64% at day 8.

#### 2.4.2. Pigment Content

**Figure 3:**
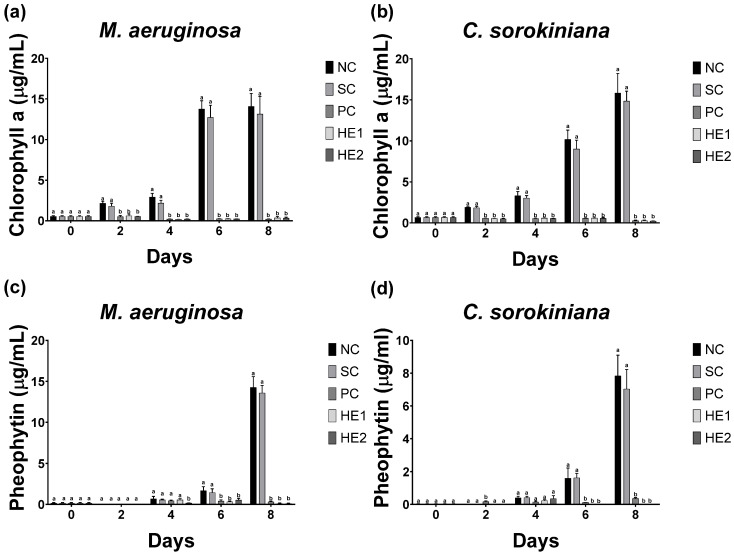
Effect of HE1 and HE2 extracts at the minimum inhibitory concentration on *M. aeruginosa* (**a**,**c**) and *C. sorokiniana* (**b**,**d**) chlorophyll *a* content (**a**,**b**), and pheophytin *a* content (**c**,**d**). Each value is the mean ± SD of three replicates. Different letters represent statistically significant differences (*p* < 0.05). NC, negative control; SC, solvent control (DMSO); PC, positive control (CuSO_4_) at the minimum inhibitory concentration.

To determine whether the synthesis of chlorophyll *a* and pheophytin *a* in treated cells was inhibited by *P. furfuracea* volatile extracts (HE1 and HE2), the chlorophyll *a* and pheophytin *a* contents of *M. aeruginosa* and *C. sorokiniana* cells after exposure to *P. furfuracea* volatile extracts are shown in Figure 3. For *M. aeruginosa*, the negative control levels of chlorophyll *a* increased gradually to a maximum of 14.06 µg/mL at day 8. However, treatment of this species with PC (CuSO_4_) and both extracts induced a significant decrease in the concentration of chlorophyll *a* compared to the control, which was visible from the second day until the last day of treatment (Figure 3a). No significant difference was noted between the negative and solvent controls, or between the positive control and treatments. Pheophytin *a* levels were very low or undetectable in all the treatments during the first two days of the experiment (Figure 3c). Nevertheless, from the fourth day, pheophytin *a* levels rose in all the treatments except for HE2 extract. They increased steadily in negative and solvent controls at days 6 and 8, reaching a maximum of 14.26 µg/mL. Pheophytin *a* levels remained low in PC and both extracts, not exceeding 0.54 µg/mL.

For *C. sorokiniana*, the concentration of chlorophyll *a* in the negative control showed the same pattern, with an increase to a maximum of 15.81 µg/mL at day 8 (Figure 3b,d). However, treatment of this species with PC (CuSO_4_) and both extracts induced a significant decrease in the concentration of chlorophyll *a* compared to the control, which was visible starting on day 2 and until the last day of treatment (Figure 3b). No significant difference was noted between the negative and solvent controls, or between the positive control and treatments with both extracts. Similar to *M. aeruginosa*, pheophytin *a* levels were very low or not detected on the first two days of the experiment (except for PC at day 2). At day 4, levels of pheophytin *a* increased in all treatments and continued to increase significantly at days 6 and 8 only for NC and SC, while they remained low for PC, HE1, and HE2.

#### 2.4.3. Total Protein Content

**Figure 4:**
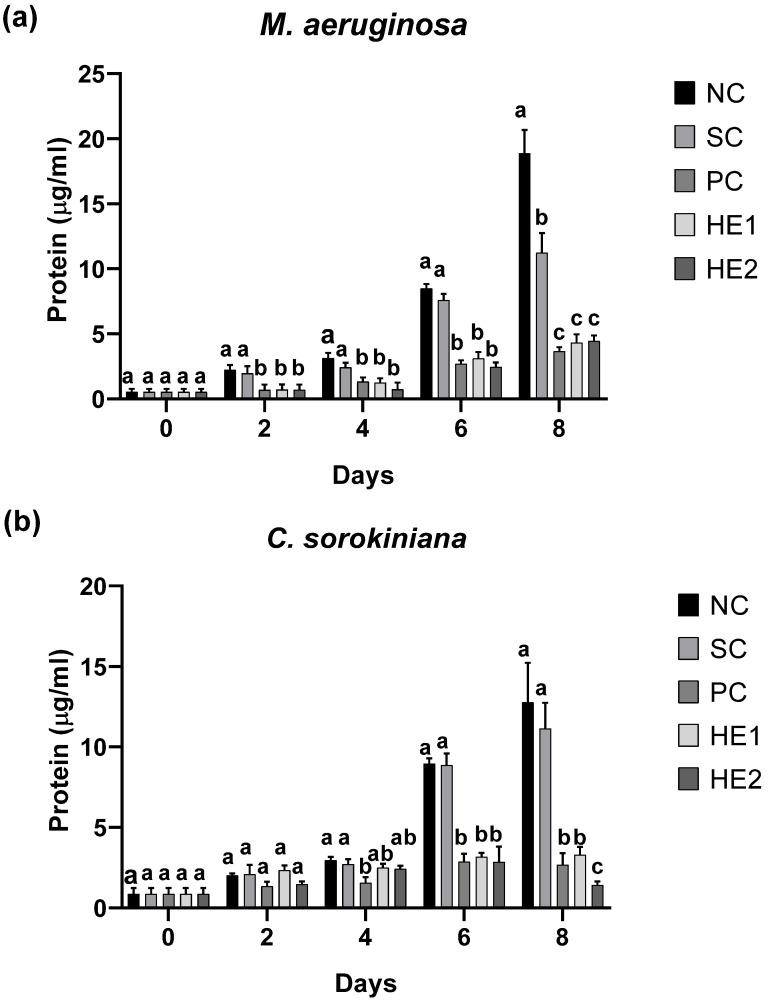
Effect of HE1 and HE2 extracts at the minimum inhibitory concentration on *M. aeruginosa* (**a**) and *C. sorokiniana* (**b**) protein contents. Each value is the mean ± SD of three replicates. Different letters represent statistically significant differences (*p* < 0.05). NC, negative control; SC, solvent control (DMSO); PC, positive control (CuSO_4_) at the minimum inhibitory concentration.

To investigate the effects of *P. furfuracea* extracts on protein synthesis in *M. aeruginosa* and *C. sorokiniana* cells, protein levels were determined after exposure to both extracts every 48 h for 8 days, and the results are shown in Figure 4. For *M. aeruginosa*, the level of proteins in the negative control increased gradually to a maximum of 18.85 µg/mL at day 8. However, treatment with PC and both extracts induced a significant decrease in the concentration of proteins compared to both negative (NC) and solvent (SC) controls, which was visible starting on day 2 and until the last day of treatment (Figure 4a). No significant difference was noted between the negative (NC) and solvent (SC) controls, except for day 8.

For *C. sorokiniana*, the concentration of proteins in the negative control (NC) showed the same pattern, with an increase to a maximum of 12.75 µg/mL at day 8 (Figure 4b). However, treatments with PC and both extracts induced a significant decrease in the concentration of proteins compared to both negative (NC) and solvent (SC) controls, which was visible starting on day 6 and until the last day of treatment. At day 8, no significant difference was observed between the negative and solvent controls; however, HE2 induced a significantly greater reduction in protein concentration than HE1.

#### 2.4.4. Superoxide Dismutase Activity

**Figure 5:**
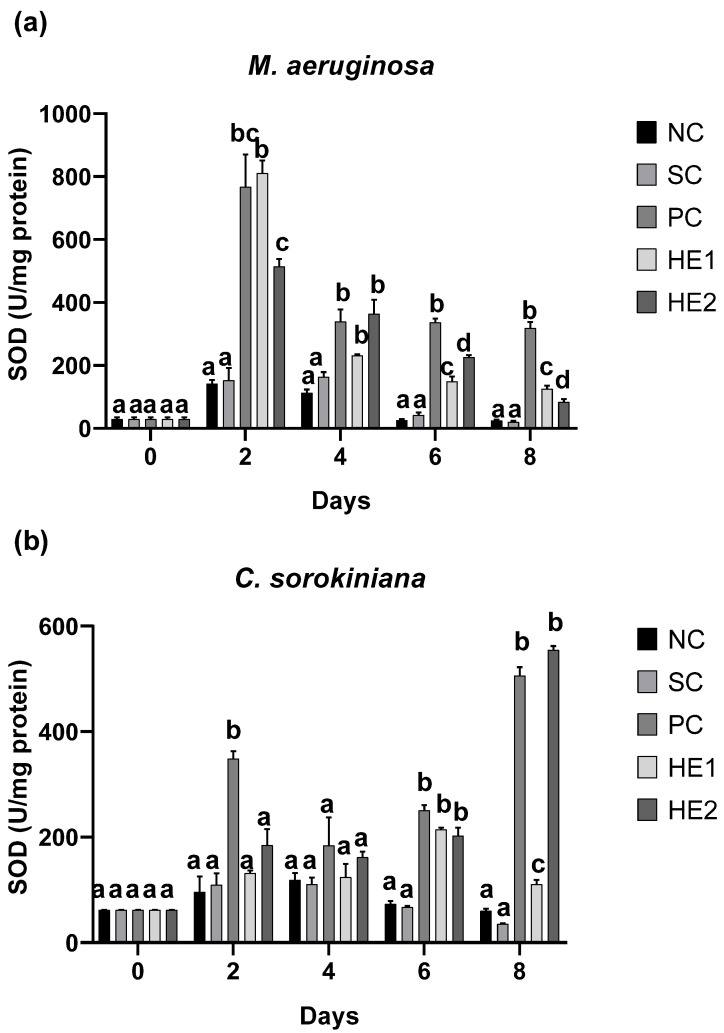
Effect of HE1 and HE2 extracts at the minimum inhibitory concentration on *M. aeruginosa* (**a**) and *C. sorokiniana*. (**b**) superoxide dismutase activity. Each value is the mean ± SD of three replicates. Different letters represent statistically significant differences (*p* < 0.05). NC, negative control; SC, solvent control (DMSO); PC, positive control (CuSO_4_) at the minimum inhibitory concentration.

The activity of the antioxidant enzyme superoxide dismutase (SOD) was investigated on *M. aeruginosa* and *C. sorokiniana* cultures to determine if HE1 and HE2 extracts affect their antioxidant defense systems (Figure 5). The results show that SOD activities in both species exposed to negative (NC) and solvent (SC) controls remained stable with no significant differences during the entire treatment period (Figure 5a,b). In comparison, a significant increase in SOD activity was visible in both *M. aeruginosa* and *C. sorokiniana* cells treated with HE1 and HE2 extracts compared to both controls and during all treatment periods for *M. aeruginosa* and only at days 6 and 8 for *C. sorokiniana*.

For *M. aeruginosa*, HE1 extract induced a significant increase in SOD activity compared to the negative control (NC) at days 2, 4, 6, and 8, with a maximum reached at day 2, followed by a gradual decrease over the following days (Figure 5a). Similarly, HE2 exhibited a similar pattern, with a maximum at day 2 and a gradual decrease on subsequent days (Figure 5a). On the other hand, the positive control (copper sulfate) induced a significant increase in SOD activity at day 2 and then had a relatively stable but moderately high value until day 8. However, in *C. sorokiniana* cells, HE1 extract induced a significant increase in SOD activity at days 6 and 8. Similarly, HE2 extract induced a significant increase in SOD activity at days 6 and 8. In contrast, the positive control (PC) varied over time, with a significant increase on days 2, 6, and 8, and values not significantly different from the controls on day 4.

#### 2.4.5. Catalase Activity

**Figure 6:**
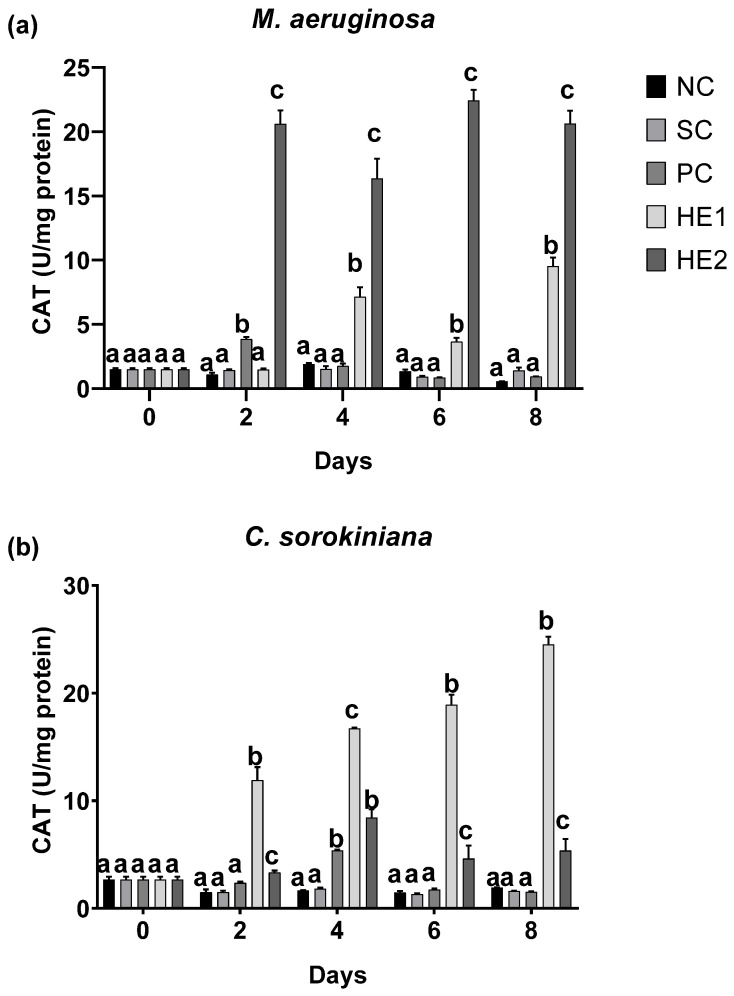
Effect of HE1 and HE2 extracts at the minimum inhibitory concentration on *M. aeruginosa* (**a**) and *C. sorokiniana* (**b**) catalase activity. Each value is the mean ± SD of three replicates. Different letters represent statistically significant differences (*p* < 0.05). NC, negative control; SC, solvent control (DMSO); PC, positive control (CuSO_4_) at the minimum inhibitory concentration.

The antioxidant catalase (CAT) enzyme is another important defense mechanism against reactive oxygen species (ROS). As shown in Figure 6, the CAT activity in *M. aeruginosa* and *C. sorokiniana* for the negative (NC) and solvent (SC) controls remained unchanged over time. For *M. aeruginosa*, HE1 extract induced a significant increase in CAT activity at days 4, 6, and 8 (Figure 6a). On the other hand, exposure to HE2 extract showed significantly higher levels of CAT activity at days 2, 4, 6, and 8 compared to HE1 extract. For the positive control (PC), with the exception of day 2, there was no significant difference compared to both negative (NC) and solvent (SC) controls in CAT activity.

For *C. sorokiniana*, HE1 extract induced a significant increase in CAT activity compared to both controls (NC, SC) and the positive control (PC) throughout the experiment (Figure 6b). On the other hand, HE2 extract also induced a significant increase in CAT activity during the experiment, but with a more modest increase than HE1. Finally, the positive control induced no significant difference in CAT activity in comparison to both controls (NC, SC), with the exception of day 4 (Figure 6b).

#### 2.4.6. Malondialdehyde Levels

**Figure 7:**
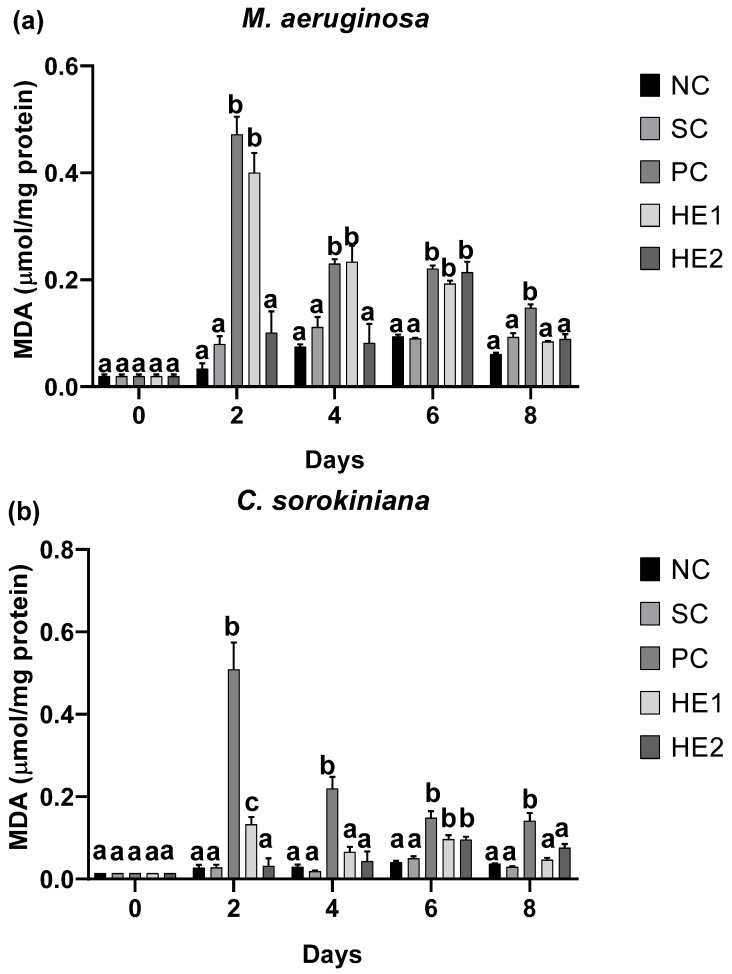
Effect of HE1 and HE2 extracts at the minimum inhibitory concentration on *M. aeruginosa* (**a**) and *C. sorokiniana*. (**b**) malondialdehyde contents. Each value is the mean ± SD of three replicates. Different letters represent statistically significant differences (*p* < 0.05). NC, negative control; SC, solvent control (DMSO); PC, positive control (CuSO_4_) at the minimum inhibitory concentration.

Malondialdehyde (MDA) levels are used as a biomarker of lipid peroxidation. The MDA levels in *M. aeruginosa* and *C. sorokiniana* cells exposed to both negative (NC) and solvent (SC) controls increased slightly but not significantly throughout the duration of the experiment (Figure 7). For *M. aeruginosa*, HE1 extract induced a significant increase in MDA levels compared to the negative control at days 2, 4, and 6, reaching a maximum at day 2 and returning to the control level at day 8 of exposure (Figure 7a). On the other hand, HE2 extract induced no significant differences with both negative (NC) and solvent (SC) controls during the treatment periods, with the exception of day 6. The positive control (PC) exhibited a pattern similar to that of HE1, with a significant increase compared to both negative (NC) and solvent (SC) controls at days 2, 4, 6, and 8, and with a maximum reached at day 2.

For *C. sorokiniana*, HE1 extract induced a significant increase in MDA levels only at days 2 and 6 (Figure 7b). Similarly, HE2 extract induced a significant increase in MDA levels only at day 6. On the other hand, the positive control (PC) induced a significant increase in MDA levels compared to both negative (NC) and solvent (SC) controls throughout the experiment, with a maximum value reached at day 2.

### 2.5. In Silico Study

#### 2.5.1. Molecular Docking

**Figure 8:**
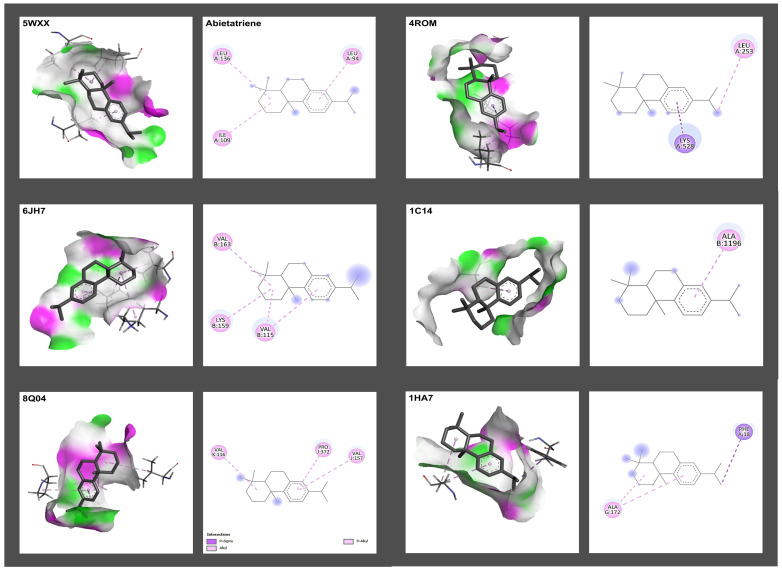
Representative docking interactions of abietatriene against six molecular targets. 5WXX: aspartate racemase, 4R0M: McyG adenylation–peptidyl carrier protein didomain, 6JH7: AerF protein, 1C14: enoyl-[acyl-carrier-protein] reductase, 8Q04: RuBisCO, 1HA7: phycocyanin. In the 3D representation, the ligand-protein complex was stabilized by a network of conventional hydrogen bonds (indicated in green) and hydrophobic interactions (indicated in pink).

**Figure 9:**
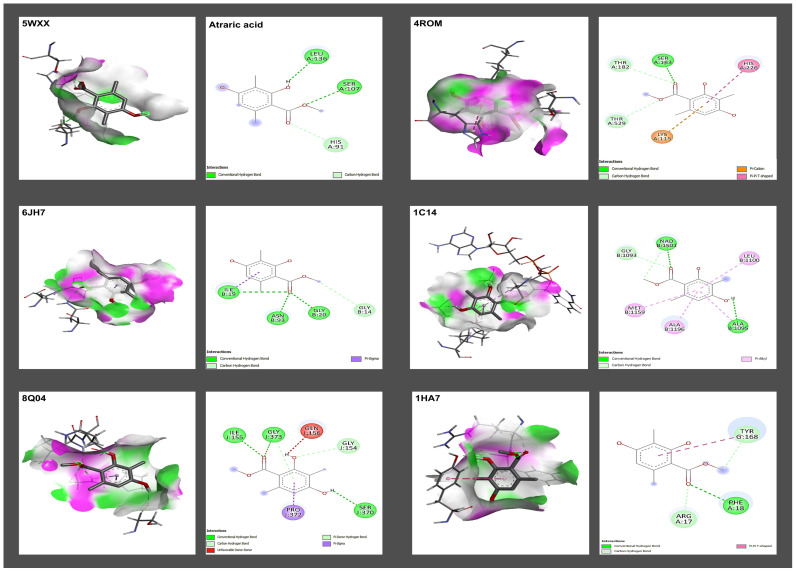
Representative docking interactions of atraric acid against six molecular targets. 5WXX: aspartate racemase, 4R0M: McyG adenylation–peptidyl carrier protein didomain, 6JH7: AerF protein, 1C14: enoyl-[acyl-carrier-protein] reductase, 8Q04: RuBisCO, 1HA7: phycocyanin. In the 3D representation, the ligand-protein complex was stabilized by a network of conventional hydrogen bonds (indicated in green) and hydrophobic interactions (indicated in pink).

**Figure 10:**
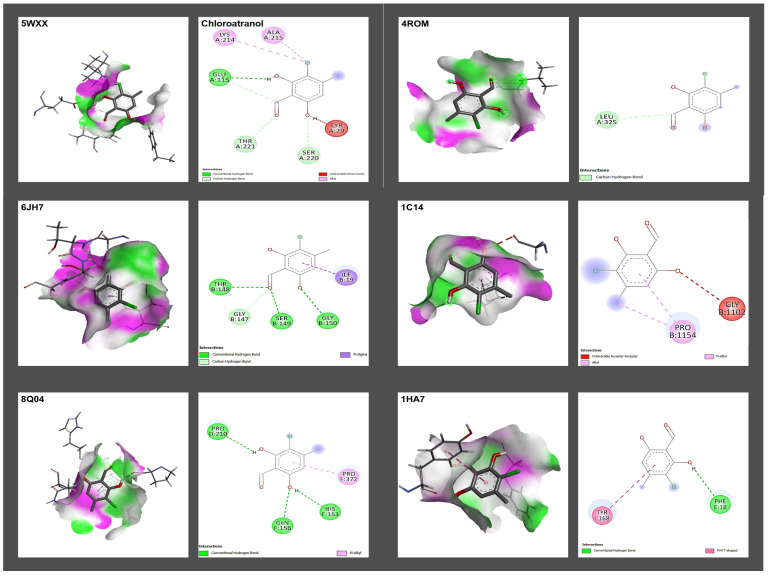
Representative docking interactions of chloroatranol against six molecular targets. 5WXX: aspartate racemase, 4R0M: McyG adenylation–peptidyl carrier protein didomain, 6JH7: AerF protein, 1C14: enoyl-[acyl-carrier-protein] reductase, 8Q04: RuBisCO, 1HA7: phycocyanin. In the 3D representation, the ligand-protein complex was stabilized by a network of conventional hydrogen bonds (indicated in green) and hydrophobic interactions, including π-alkyl and π - π stacking configurations (indicated in pink).

Molecular docking analysis was performed to predict the in silico binding orientation and stability between a ligand and a target protein. This interaction is quantified by the binding energy (expressed in kcal/mol), where a lower (more negative) energy value indicates a higher binding affinity and a more stable interaction. Results presented in Table 6 and Figure 8, Figure 9 and Figure 10 illustrate binding energies and docking interactions for some selected lichen volatile compounds ranging from −3.9 to −8.4 kcal/mol. The diterpene abietatriene, identified in this study only in the extract HE2 (Table 1), demonstrated the strongest binding across multiple proteins (−8.3 to −8.4 kcal/mol), followed by guaiol acetate (−6.3 to −7.5 kcal/mol) and δ-cadinene (−6.1 to −7.5 kcal/mol), both identified only in the extract HE1 (Table 1).

Moderately strong interactions were observed for atraric acid (−6.1 to −7.2 kcal/mol) and chloroatranol (−4.7 to −6.3 kcal/mol), both of which are major constituents of the two extracts HE1 and HE2 (Table 1). Conversely, weaker binding was associated with 2-furancarboxaldehyde and long-chain fatty acids (−3.9 to −5.8 kcal/mol). Representative 2D and 3D interaction diagrams (Figure 8, Figure 9 and Figure 10) illustrate these binding modes, specifically for Abietatriene (Figure 8) with aspartate racemase (5WXX) and atraric acid (Figure 9) with AerF (6JH7), highlighting the key hydrogen bonding and hydrophobic contacts within the active sites.

#### 2.5.2. Absorption, Distribution, Metabolism, and Excretion (ADME)

As illustrated in Table 7, SwissADME profiling showed that most compounds complied with Lipinski’s, Veber’s, and Egan’s rules, suggesting acceptable oral bioavailability. Exceptions included long-chain fatty acids and resin acids (e.g., n-hexadecanoic acid, abietatriene, 9,12,15-octadecatrienoic acid), which exceeded recommended lipophilicity thresholds.

Furthermore, absorption was predicted to be high for the majority of molecules, though naphthalene, δ-cadinene, and some fatty acids showed low gastrointestinal uptake. Blood–brain barrier permeability was indicated for small lipophilic molecules (e.g., trans-verbenol, naphthalene, quinoline), while larger oxygenated terpenes were predicted to be non-permeant.

Additionally, no compounds triggered potential assay interference and structural liabilities (PAINS) alerts; however, Brenk filters flagged potential reactive groups, notably aldehydes (chloroatranol, methyl hematommate) and phenols (acetisoeugenol). Several molecules were predicted to inhibit major cytochrome P450 isoforms (CYP1A2, CYP2C19, CYP2C9, CYP3A4), suggesting possible drug–drug interaction liabilities.

**Figure 11:**
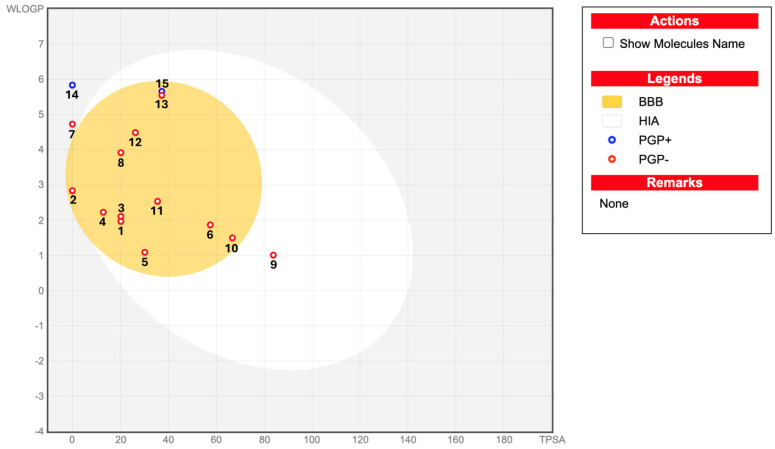
Boiled-egg graph of the 15 phytoconstituents from the lichen *Pseudevernia furfuracea*. 1: Trans-verbenol, 2: Naphthalene, 3: ρ-Cymene-8-ol 4: Quinoline, 5: 2-Furancarboxaldehyde, 6: Chloroatranol, 7: δ-Cadinene, 8: Himachalol, 9: Methyl hematommate, 10: Atraric acid, 11: Acetisoeugenol, 12: Guaiol acetate, 13: n-Hexadecanoic acid, 14: Abietatriene, 15: 9,12,15-Octadecatrienoic acid.

**Figure 12:**
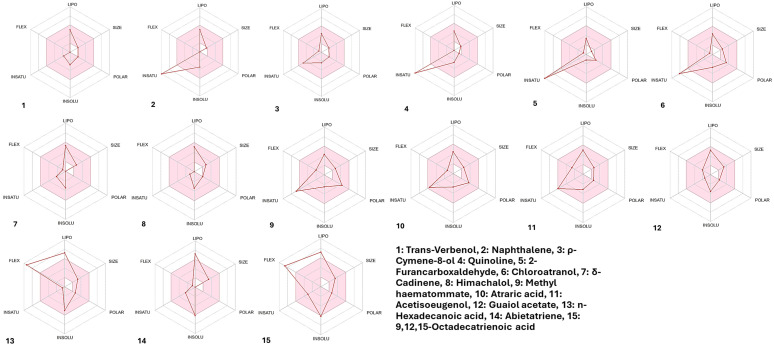
Bioavailability radar plot of the 15 phytoconstituents from the lichen *Pseudevernia furfuracea*.

Skin permeability predictions indicated higher values for fatty acids compared with small aldehydes, while P-gp substrate analysis suggested efflux risk for long-chain lipophilic molecules. The boiled-egg model (Figure 11) and radar plots (Figure 12) further illustrated favorable drug-likeness for atraric acid, guaiol acetate, δ-cadinene, and trans-verbenol.

#### 2.5.3. Toxicity Predictions

ProTox-III classified most compounds into toxicity classes IV–VI, indicating relatively low acute oral toxicity (Table 8). Predicted LD_50_ values ranged from 65 mg/kg for 2-furancarboxaldehyde (class III, highly toxic) to 10,000 mg/kg for 9,12,15-octadecatrienoic acid (class VI, practically non-toxic).

Organ-specific predictions showed hepatotoxicity alerts for acetisoeugenol, while carcinogenicity was flagged for naphthalene, ρ-cymene-8-ol, and 2-furancarboxaldehyde. Mutagenicity was predicted for quinoline and 2-furancarboxaldehyde, whereas immunotoxicity risks were moderate for acetisoeugenol and himachalol. Cytotoxicity predictions were generally negative across most compounds.

## 3. Discussion

The present study demonstrates that extracts (HE1 and HE2) from the lichen *Pseudevernia furfuracea* containing volatile compounds exhibit potent microbicidal and algicidal activity against *Microcystis aeruginosa* and *Chlorella sorokiniana*. While the anti-cyanobacterial and anti-microalgal potential of non-volatile lichen secondary metabolites has been previously documented [20,22], this work establishes that lichen volatile compounds also possess significant inhibitory properties against ecologically relevant bloom-forming strains.

Quantitative evaluation revealed robust inhibitory potential, with minimum inhibitory concentrations (MIC) reaching 375 µg/mL. HE1 extract exhibited a lower MIC against the cyanobacterium *M. aeruginosa* (375 µg/mL) compared to the eukaryotic microalga *C. sorokiniana* (750 µg/mL), suggesting a degree of selective toxicity. Because there are no prior studies on lichen volatiles against these specific organisms, direct comparisons must be drawn from broader allelochemical research. Our observed MICs are highly competitive when compared to other plant-derived volatiles; for instance, essential oils from *Thymus* species and *Cystoseira tamariscifolia* required much higher concentrations (up to 5 mg/mL) to achieve similar growth inhibition in *M. aeruginosa* [25,26]. The efficacy of HE1 and HE2 extracts likely stems from their lipophilic terpenoid composition, which enables cell lysis through membrane disruption, a mechanism well-documented for volatile allelochemicals in aquatic systems.

The translation of this inhibitory capacity into altered growth dynamics was striking. Treatments with the two extracts (HE1, HE2) induced a near-total collapse of cell density (up to 98% inhibition by day 8), comparable to the algaecide copper sulfate used as a positive control. However, the physiological response differed markedly between the two strains. In *M. aeruginosa*, the growth rate was severely slowed, whereas in *C. sorokiniana*, the growth rate became strictly negative. This divergence points to distinct mechanisms of action governed by the cellular architecture of the two models.

These growth disruptions were intimately linked to the collapse of primary metabolic functions, evidenced by significant reductions in chlorophyll *a* and total protein content. The parallel decline in these parameters suggests that HE1 and HE2 extracts either directly inhibit photosynthetic protein synthesis or trigger widespread cellular degradation [27]. Because we did not observe a proportional increase in pheophytin *a*, a primary catabolite of chlorophyll *a*, the data strongly suggest that the volatile compounds primarily suppress *de novo* chlorophyll *a* synthesis rather than solely accelerating its enzymatic degradation. This aligns with the findings of Bačkor et al. [20], who demonstrated that lichen metabolites like usnic acid disrupt pigment composition and halt photobiont proliferation by interfering with the photosynthetic apparatus.

To elucidate the mechanisms driving this metabolic collapse, we assessed oxidative stress responses and lipid peroxidation. The production of reactive oxygen species (ROS) is a primary hallmark of allelochemical toxicity in aquatic organisms. Treatment with HE1 and HE2 extracts triggered significant elevations in superoxide dismutase (SOD) and catalase (CAT) activities, though the timing and intensity varied distinctly between the two species. In the cyanobacterium *M. aeruginosa*, SOD activity spiked rapidly (by day 2) and was accompanied by a severe accumulation of malondialdehyde (MDA) when treated with the extract HE1. This profile closely mirrors the effects of copper sulfate and glyphosate [28,29], indicating that HE1 extract induces acute oxidative stress that overwhelms the cyanobacterial defense systems, leading to extensive lipid peroxidation and catastrophic membrane disruption.

Conversely, the eukaryotic green alga *C. sorokiniana* exhibited a markedly different physiological response. Despite experiencing a complete cessation of growth (negative growth rate), the induction of antioxidant enzymes was delayed until day 6, and MDA levels did not exhibit the catastrophic spikes seen in *M. aeruginosa*. This paradox, severe growth inhibition without acute, non-specific membrane destruction, suggests that lichen volatile compounds operate via highly targeted, non-lytic mechanisms against green microalgae. This finding holds profound ecological significance. It strongly supports the hypothesis that lichen secondary metabolites function as precise allelochemical regulators within the symbiotic thallus, designed to suppress photobiont overgrowth and manage partner selection without destroying the host’s photosynthetic machinery [16,30].

To map these physiological observations to specific molecular interactions, we integrated molecular docking with ADMET predictions. Rather than acting through a single pathway, the bioactivity of HE1 and HE2 extracts is likely driven by a synergistic combination of highly affine terpenoids and abundant phenolic compounds. Docking simulations identified hydrophobic terpenoids, specifically abietatriene, guaiol acetate, and δ-cadinene, as the most potent binders against key microbial and cyanobacterial targets, including AerF (6JH7) and the microcystin synthetase protein McyG (4R0M). The rigid hydrophobic scaffolds of these terpenoids allow them to anchor deeply within non-polar protein pockets, disrupting essential enzymatic functions.

While these highly affine diterpenes provide potency, the overall efficacy of the volatile fractions is heavily supported by the sheer abundance of phenolic derivatives like atraric acid and chloroatranol. Although these abundant metabolites demonstrated moderate binding affinities compared to the terpenoids, their high concentration ensures persistent target saturation. This balance between binding strength and compound abundance is a defining characteristic of complex bioactive mixtures.

Finally, applying the ADMET framework to an ecological context provides a compelling mechanistic model for how these volatile compounds function in aquatic environments. In this context, “absorption” effectively models the penetration of these lipophilic compounds through the cyanobacterial peptidoglycan layer or the microalgal cell wall. The use of these pharmacological tools is justified by the fundamental requirement for allelochemicals to partition from the aqueous phase and permeate lipid bilayers to reach intracellular targets. While ADMET is traditionally human-focused, parameters such as Log P and the boiled-egg model serve as models that may predict how these metabolites interact with the cellular envelopes of *M. aeruginosa* and *C. sorokiniana*. This approach is consistent with recent studies demonstrating that the phytotoxic activity of volatile components is fundamentally governed by their molecular structure and their specific affinity for membrane lipid interactions [31]. Highly lipophilic resin acids, while showing strong in silico docking, exhibited poor predicted solubility and high efflux potential, suggesting limited bioavailability in aqueous systems. In contrast, oxygenated terpenoids and phenolic esters displayed the optimal balance of moderate affinity and favorable environmental pharmacokinetics (e.g., ability to cross membranes and accumulate in photosynthetic complexes). Toxicity predictions further corroborated our biochemical findings, linking these specific volatile classes to the disruption of electron transport and the induction of oxidative stress.

Ultimately, this integrative approach demonstrates that lichen volatile compounds are not merely non-specific toxins. They are a complex suite of highly specialized metabolites that, through targeted molecular interactions and favorable lipophilic properties, selectively disrupt photosynthetic and metabolic pathways, making them highly promising candidates for the eco-friendly management of harmful algal and cyanobacterial blooms.

## 4. Materials and Methods

### 4.1. Lichen Material Sampling and Extraction

*Pseudevernia furfuracea* var. *furfuracea* specimens were harvested in June 2021 from two Moroccan habitats: the High Atlas (on *Quercus rotundifolia*; 31°28′19.5″ N 7°25′27.6″ W, 1644 m) and the Middle Atlas (on *Cedrus atlantica*; 33°25′2″ N 5°11′10″ W, 1776 m). Taxonomic authentication was performed by co-author Y.E. using morphological keys and spot tests (cortex K−/C−, medulla K+/C−), with voucher specimens (voucher numbers 9 and 15) deposited in herbaria. The two volatile extracts HE1 and HE2 were prepared from two specimens of *P. furfuracea* var. *furfuracea* collected from the High Atlas and Middle Atlas Mountains, respectively. Briefly, 100 g of shade-dried lichen was ground and extracted by hydrodistillation for 4 h, according to the protocol of Sanad, et al. [32]. The distillate was then extracted with dichloromethane, and after evaporation of the solvent, the resulting extracts were stored at −20 °C for various analyses.

Chemical profiling of HE1 and HE2 was previously published in Essadki, et al. [24]. It was performed using a QExactive Quadrupole-Orbitrap GC-MS (Thermo Fisher Scientific, Waltham, MA, USA) at 60,000 resolution. Separation utilized an Agilent capillary column (30 m × 0.25 mm, 0.25 µm) with helium (1 mL/min) as the carrier gas. The oven program started at 60 °C (1 min), increased at 5 °C/min to 210 °C, then at 10 °C/min to 280 °C (15 min hold). Injection was in split mode (1:50) at 250 °C with EI ionization at 70 eV (full scan, *m*/*z* 35–650). Data were processed via Xcalibur 4.3 and Compound Discoverer™ 3.2, with identification confirmed against the NIST library and retention indices. Quantification was achieved through peak area normalization.

### 4.2. Isolation and Culture Conditions of the Cyanobacterial and Microalgal Strain

For the cyanobacteria strain, a unicellular strain of *Microcystis aeruginosa* was previously isolated from a bloom sampled in October 2015 in the Lalla Takerkoust lake reservoir (31°21′36″ N; 8°7′48″ W) near Marrakech, Morocco. Details of the identification, isolation, separation into single cells, and maintenance procedure for *M. aeruginosa* were determined in accordance with the protocol described in Zerrifi, et al. [33].

As for the microalgae strain, a 500 mL water sample was collected from a basin within the Faculty of Sciences Semlalia of Marrakech (31°39′ N, 8°00′56″ W). The sample was concentrated by successive centrifugations and plated on solid Z8 medium. To obtain pure isolates, repeated sub-culturing was performed. Colonies with a macroscopic appearance characteristic of *Chlorella*, confirmed microscopically as *Chlorella sorokiniana* Shihira & R.W. Krauss 1965, were selected and maintained in batch cultures of Z8 liquid medium.

Both strains were thereafter maintained in a controlled chamber at 25 ± 0.6 °C, under a light intensity of 60 µmol photons·m^−2^·s^−1^, with a 15 h/9 h light/dark cycle.

### 4.3. Screening for Algicidal Activity

#### 4.3.1. Disc Diffusion Method

A qualitative assessment of HE1 and HE2’s algicidal potential was carried out on solid medium using a modified double-layer agar plate method, following Uchida, et al. [34]. Briefly, 20 mL of Z8 medium supplemented with 1.2% (*w*/*v*) agar was poured into 90 mm Petri dishes and overlaid with 4 mL of soft agar (0.9%) containing 2 mL of *M. aeruginosa* or *C. sorokiniana* in the exponential growth phase. After solidification, 9 mm sterile Whatman discs loaded with 20 µL of HE1 or HE2 (1 mg/mL in dimethylsulfoxide, DMSO) were placed on the agar surface. An aliquot of 20 µL of copper sulfate (30 mg/mL) served as a positive control, and 20 µL of DMSO as a negative control. Plates were held at 4 °C for 4 h to allow diffusion, then incubated for 8 days under the culture conditions already mentioned above. After incubation, inhibition zones were measured with a caliper. Each treatment was tested in triplicate, and the experiment was independently repeated three times.

#### 4.3.2. Broth Microdilution Assay

The minimum inhibitory concentrations (MICs) of HE1, HE2, copper sulfate, and DMSO were determined using the broth microdilution method described by Zerrifi et al. [28]. Briefly, in a 96-well microplate, serial dilutions of HE1 and HE2 were prepared in Z8 medium supplemented with 1% DMSO, at a final volume of 100 µL across 12 wells (concentrations ranging from 1500 to 0.73 µg/mL), per well. Then, 100 µL of a suspension of the test organisms (*M. aeruginosa* or *C. sorokiniana*) in the exponential growth phase, adjusted to 4 × 10^6^ cells/mL, was added to each well, giving a final volume of 200 µL. Each plate included a solvent control (Z8 medium with 1% DMSO), a positive control (copper sulfate 30 mg/mL), and a growth control (Z8 medium with microorganism only). Plates were incubated for 8 days under the described controlled conditions of the culture chamber. The lowest concentration of the tested substance with no visible growth was recorded as the MIC. Subsequently, an aliquot of 100 µL from wells showing no visible growth was plated on Z8 medium to determine the minimum microbicidal concentration (MMC). All assays were performed in triplicate and repeated independently three times.

### 4.4. Growth Monitoring and Biochemical Parameters

#### 4.4.1. Determination of the Growth Parameters

The physiological effects of HE1 and HE2 extracts on *M. aeruginosa* and *C. sorokiniana* were studied following the protocol described in Zerrifi et al. [28]. Briefly, Erlenmeyer flasks containing 100 mL of Z8 medium inoculated at an initial density of 2 × 10^6^ cells/mL (exponential growth phase) of *M. aeruginosa* and *C. sorokiniana* strains were treated with HE1 and HE2 at their respective MICs and incubated at 25 ± 0.6 °C, under a light intensity of 60 µmol photons·m^−2^·s^−1^, with a 15 h/9 h light/dark cycle for 8 days. The 8-day experimental period was selected to ensure that both *M. aeruginosa* and *C. sorokiniana* reached their late exponential growth phases under the defined culture conditions. This duration allows for the observation of long-term physiological impacts and helps distinguish between transient growth delays (microbiostatic effects) and permanent collapse (microbicidal effects), consistent with established protocols for assessing allelopathic interactions in phytoplankton [28,33]. Copper sulfate (MIC) serving as the positive control, 1% DMSO as the solvent control, and untreated cultures serving as the negative control were incubated in the same conditions. Cell counts were performed every 48 h using a Malassez counting chamber, according to Sbiyyaa, et al. [35]. In brief, the chamber was filled with the sample and allowed to stand for 10 min to ensure sedimentation. At least 10 squares (0.01 µL each) were counted, and the mean cell number was calculated.

Algal density (cells/mL) was calculated as:

(1) $$\mathrm{Algal}\;\mathrm{density}\;\left(\mathrm{cells}/\mathrm{mL}\right)\;=\;\bar{N}\;\times \;10^{5}$$

with $\bar{N}$ being the mean number of cells in 10 squares of 0.01 µL.

The inhibition rate (IR, %) was determined relative to the negative control using:

(2) $$IR\;(\%)\;=\;\frac{N_{control}\;-\;N_{treatment}}{N_{control}}\;\times \;100$$

where $N_{control}$ and $N_{treatment}$ represent cell concentrations in the control and treated samples, respectively.

The growth rate (µ) was calculated according to Xu, et al. [36]:

(3) $$µ\;=\;\frac{ln(N_{t}/N_{0})}{\Delta t}$$

where $N_{t}$ and $N_{0}$ are the cell densities (cells/mL) at the last and first day of the assay, respectively, and Δt is the assay duration.

#### 4.4.2. Determination of Chlorophyll *a* and Pheophytin *a* Contents

Chlorophyll *a* and pheophytin *a* concentrations were measured spectrophotometrically every 48 h according to Yéprémian, et al. [37] (SOP 2.3, SOP 2.4). Briefly, 2 mL of culture was centrifuged at 4000× *g* for 15 min at 4 °C to pellet algal cells. Ten milliliters of ethanol were added, mixed thoroughly, and heated to 75 °C. The mixture was sonicated for 10 min in an ultrasonic bath and centrifuged. The supernatant absorbance was then recorded at 665 and 750 nm, using ethanol as a blank. For pheophytin *a* quantification, 20 µL of 1 M HCl was added to the ethanol extract and incubated for 2 min, then neutralized with 20 µL of 1 M NaOH and incubated for 2 min before re-reading at 665 and 750 nm.

Chlorophyll *a* and pheophytin *a* concentrations (µg/L) were calculated using the following equations:

(4) $${Chl}_{a}\;=\;\frac{28.44\;\times \;(Abs665b\;-\;Abs665a)\;\times \;Ve}{V_{s}\;\times \;I}$$

(5) $$Pheophytin\;a\;=\;\frac{20.47\;\times \;Abs665a\;\times \;Ve}{Vs\;\times \;I}\;-\;{Chl}_{a}$$

where Ve is the ethanol volume (mL), $V_{s}$ is the sample volume (L), and I is the cuvette path length (cm). Abs665b refers to the absorbance at 665 nm before acidification, and Abs665a to the absorbance at 665 nm after acidification. Absorbances were corrected by subtracting the corresponding value at 750 nm.

#### 4.4.3. Preparation of Enzyme Extract and Determination of the Total Protein Content

Antioxidant enzyme extracts were prepared following Li, et al. [38]. Every two days, 2 mL of suspension was sampled from each Erlenmeyer flask and centrifuged at 4000× *g* for 10 min at 4 °C. The pellet was resuspended in 0.1 M phosphate buffer (pH 6.5) supplemented with 1% (*w*/*v*) polyvinylpyrrolidone (PVP). Cells were disrupted by sonication in an ice bath for 5 min and centrifuged again. The resulting supernatant was used as an enzyme extract.

Total protein content was determined using the Bradford [39] method. Briefly, 100 µL of enzyme extract was mixed with 1 mL of Bradford reagent and incubated in the dark at room temperature (25 ± 2 °C) for 20 min. Absorbance was measured at 595 nm using an Agilent Cary 50 Scan UV-Vis spectrophotometer (Agilent Technologies, Santa Clara, CA, USA), with buffer plus Bradford reagent as a blank. Protein concentration was calculated from a standard curve of bovine serum albumin (BSA). Total protein content per treatment was used to normalize the specific activity of antioxidant enzymes.

#### 4.4.4. Determination of Superoxide Dismutase (SOD) Activity

Superoxide dismutase (SOD) activity was determined according to Beauchamp and Fridovich [40]. The reaction mixture contained 0.8 mL phosphate buffer saline (PBS, 50 mM, pH 7.8), 0.3 mL methionine (130 mM), 0.3 mL Na_2_EDTA (100 µM), 0.3 mL riboflavin (20 µM), 0.3 mL nitro blue tetrazolium (NBT, 750 µM), and 1 mL enzyme extract. The assay was based on the inhibition of NBT photoreduction by SOD. Negative controls consisted of tubes wrapped in aluminum foil to simulate complete darkness (no photoreduction). Positive controls were exposed to light without SOD activity, allowing complete photoreduction of NBT. Treatment groups were exposed to light in the presence of enzyme extracts, where SOD activity inhibited NBT reduction. After 20 min of irradiation at 40–60 µmol photons·m^−2^·s^−1^, absorbance was measured at 560 nm. One unit of SOD activity was defined as the enzyme amount required to inhibit 50% of NBT photoreduction.

#### 4.4.5. Determination of Catalase (CAT) Activity

Catalase (CAT) activity was determined according to Rao, et al. [41]. The reaction mixture contained 0.5 mL H_2_O_2_ (0.3%), 0.5 mL distilled water, and 0.5 mL enzyme extract. Measurements were performed at room temperature (25 ± 2 °C) in a 3 mL quartz cuvette. The reaction was initiated by adding H_2_O_2_ and mixing with a glass rod, and absorbance was monitored at 240 nm every 30 s for 5 min using an Agilent Cary 50 Scan UV-Vis spectrophotometer. One unit of CAT activity was defined as the amount of enzyme catalyzing the decomposition of 1 mM H_2_O_2_ per minute.

#### 4.4.6. Determination of Malondialdehyde (MDA) Content

Lipid peroxidation was assessed by quantifying malondialdehyde (MDA) using the thiobarbituric acid (TBA) assay described by Hodges, et al. [42]. Every 48 h, 0.5 mL of algal and cyanobacterial suspension culture was sampled from each Erlenmeyer and centrifuged at 4000× *g* for 20 min at 4 °C. The pellet was resuspended in 2 mL trichloroacetic acid (10%), mixed thoroughly, and centrifuged again. Two milliliters of thiobarbituric acid (0.6%) were added to the supernatant, and the mixture was heated to 95 °C for 30 min. The reaction was stopped by cooling on ice. After centrifugation (4000× *g*, 10 min, 4 °C), the absorbance of the supernatant was measured at 450, 532, and 600 nm.

MDA content (µmol/L) was calculated according to Equation (6) and results were expressed as µmol/L/mg of protein:

(6) $$\mathrm{MDA}\;=\;6.45\;\times \;({OD}_{532}\;-\;{OD}_{600})\;-\;0.56\;\times \;{OD}_{450}$$

### 4.5. Molecular Docking and ADMET Profiling

#### 4.5.1. Molecular Docking

In this study, the proteins 1HA7 and 1C14 were selected as molecular docking targets to evaluate the potential inhibitory effects of the lichen volatile compounds against *Microcystis aeruginosa* and *Chlorella sorokiniana*. Since few experimentally resolved protein structures are currently available for these organisms in the Protein Data Bank (PDB), homologous proteins from other organisms were used as surrogates. The 1HA7 structure represents the D1/D2 heterodimer of Photosystem II, a core photosynthetic complex resolved from the cyanobacterium *Spirulina platensis*, which plays a central role in light harvesting and electron transport [43]. In parallel, 1C14 corresponds to the enoyl-[acyl-carrier-protein] reductase (FabI) from *Escherichia coli*, an essential enzyme in the fatty acid biosynthesis pathway responsible for maintaining membrane integrity and cellular function [44]. The crystal structure of RuBisCO (ribulose-1,5-bisphosphate carboxylase/oxygenase) from *Chlorella sorokiniana*, the essential enzyme of the Calvin cycle that fixes CO_2_ during photosynthesis, is represented by the protein 8Q04 [45]. As for *Microcystis aeruginosa,* we selected the crystal structures of three proteins, aspartate racemase (PDB ID: 5WXX), AerF protein (PDB ID: 6JH7), and the McyG adenylation–peptidyl carrier protein (A-PCP) didomain (PDB ID: 4R0M), as molecular docking targets. Aspartate racemase (5WXX) catalyzes the reversible conversion of L-aspartate to D-aspartate, a PLP-independent reaction that plays a critical role in amino acid metabolism and contributes to the biosynthesis of microcystin precursors [46]. AerF (6JH7) functions as an NADPH-dependent alkenal double-bond reductase in the biosynthesis of the Choi moiety of aeruginosins, bioactive cyanobacterial peptides with unique mechanistic features within the short-chain dehydrogenase/reductase family [47]. Lastly, the McyG A-PCP didomain (4R0M) initiates microcystin biosynthesis by loading the starter unit for Adda residue assembly, an essential step in the non-ribosomal peptide synthetase/polyketide synthetase (NRPS/PKS) pathway, and structural insights suggest how substrate binding and domain interactions shape its catalytic cycle [48]. These proteins were chosen because they span key functional pathways: primary amino acid metabolism, toxin-related secondary metabolism, and specialized peptide biosynthesis—all of which are fundamental to the ecology, survival, and toxin production capability of *M. aeruginosa*, making them highly relevant for evaluating the modulatory effects of the lichen volatile compounds.

Briefly, protein structures relevant to *Microcystis aeruginosa* and *Chlorella sorokiniana* were retrieved from the Protein Data Bank (PDB) [49]. Selected targets included aspartate racemase (5WXX), AerF protein (6JH7), McyG adenylation–peptidyl carrier protein didomain (4R0M), enoyl-[acyl-carrier-protein] reductase (FabI, 1C14), RuBisCO (8Q04), and phycocyanin (1HA7). Proteins were prepared in Discovery Studio 2021 by removing water molecules, adding hydrogen atoms, and optimizing geometry.

Volatile compound structures were retrieved from the PubChem database [50] in SDF format (accessed 10 July 2025). Ligand preparation, including energy minimization (universal force field, charge assignment) and conversion to PDBQT format, was performed in PyRx with the embedded Open Babel tool [51].

Furthermore, molecular docking was conducted using AutoDock Vina 1.1.2 implemented in PyRx. Default exhaustiveness and grid parameters centered on the active sites of each protein were applied. Binding affinities were recorded in kcal/mol. Docking poses and ligand–protein interactions were visualized in Discovery Studio 2021 (2D and 3D representations).

#### 4.5.2. ADMET and Toxicity Predictions

Drug-likeness and pharmacokinetic parameters (absorption, distribution, metabolism, excretion) were predicted using the SwissADME web server [52] (accessed 12 August 2025). To assess oral bioavailability, Lipinski’s, Veber’s, and Egan’s rules were applied [52,53,54,55]. Pan-assay interference compounds (PAINS) and Brenk structural alerts were also evaluated [56,57].

Toxicity profiling was performed using the ProTox-III server [58] (accessed 20 August 2025). Parameters included acute oral toxicity (LD_50_, toxicity class), organ toxicity (hepatotoxicity), and toxicological endpoints (cytotoxicity, mutagenicity, carcinogenicity, immunotoxicity).

### 4.6. Statistical Analysis

Results were expressed as mean ± standard deviation (SD). Each experiment was performed in triplicate. Data normality was assessed using the Shapiro–Wilk and Kolmogorov–Smirnov tests. Differences between groups were analyzed by one-way analysis of variance (ANOVA), followed by Tukey’s honest significant difference (HSD) post hoc test. Statistical analyses were conducted with SPSS software (version 21.0). Graphs were generated using GraphPad Prism (version 8.0).

## 5. Conclusions

This study demonstrates that volatile fractions from the lichen *P. furfuracea* exert potent inhibitory effects on the growth of *M. aeruginosa* and *C. sorokiniana*. While both treatments successfully reduced chlorophyll *a* and protein levels, their physiological impacts diverged significantly between the two species. *M. aeruginosa* experienced acute oxidative stress and extensive membrane damage, whereas the eukaryotic microalga *C. sorokiniana* exhibited a non-lytic suppression of growth, highlighting a remarkable cellular resilience.

Molecular docking and ADMET predictions suggest that these divergent activities are driven by a complex interplay of lipophilic terpenoids (abietatriene, guaiol acetate, δ-cadinene) and abundant phenolic compounds (atraric acid, chloroatranol). By targeting key photosynthetic and metabolic protein complexes, these volatile compounds effectively disrupt cellular function while maintaining favorable environmental pharmacokinetics.

Ultimately, these findings indicate that lichen volatile compounds operate through both broad oxidative disruption and highly targeted metabolic suppression. This dual mechanism underscores their profound ecological role in regulating symbiotic photobionts while simultaneously positioning them as promising, eco-friendly candidates for the biocontrol of harmful algal and cyanobacterial blooms. Future research should prioritize the isolation of these key bioactive constituents and the evaluation of their efficacy and toxicological safety in complex aquatic ecosystems.

## Figures and Tables

**Table 1:** Chemical composition of the volatile fractions from *Pseudevernia furfuracea* (L.) Zopf from two different locations in Morocco obtained by GC-MS. HE1, *Pseudevernia furfuracea* volatile fraction from the High Atlas region. HE2, *Pseudevernia furfuracea* volatile fraction from the Middle Atlas region. Adapted from Essadki, et al. [24].

				Relative Abundance (%)	
Name	Formula	*m*/*z*	Retention Time (min)	HE1	HE2
Trans-verbenol	C_10_H_16_O	150.10434	10.819	-	2.25
Naphthalene	C_10_H_8_	128.06248	11.906	1.23	2.2
ρ-Cymene-8-ol	C_10_H_14_O	150.10434	11.929	-	3.04
Quinoline	C_9_H_7_N	129.05771	14.070	0.25	-
2-Furancarboxaldehyde, 5-(2-furanylmethyl)-	C_10_H_8_O_3_	176.04712	19.878	0.89	2.88
Chloroatranol	C_8_H_7_ClO_3_	186.00767	20.039	19.80	24.38
δ-Cadinene	C_15_H_24_	204.18764	23.527	1.41	-
Himachalol	C_15_H_26_O	204.18767	23.825	0.27	-
Methyl hematommate	C_10_H_10_O_5_	210.05272	24.176	0.87	-
Atraric acid	C_10_H_12_O_4_	196.0733	24.978	73.53	56.95
Acetisoeugenol	C_12_H_14_O_3_	238.08397	25.993	-	3.77
Guaiol acetate	C_17_H_28_O_2_	204.18764	26.634	0.82	-
n-Hexadecanoic acid	C_16_H_32_O_2_	256.24017	30.200	0.94	2.67
Abietatriene	C_20_H_30_	270.23474	31.946	-	1.62
9,12,15-Octadecatrienoic acid, (Z,Z,Z)-	C_18_H_30_O_2_	278.22421	33.130	-	0.18
**Total**				100	100

**Table 2:** Anti-cyanobacterial and anti-microalgal activity of HE1 (400 µg) and HE2 (400 µg) in solid medium. IZD: Inhibition zone diameter in mm. Positive control (copper sulfate at 600 µg). Solvent control at 20 µL (DMSO). Values are expressed as mean ± SD (*n* = 9). Different superscript letters within the same row indicate statistically significant differences (*p* < 0.005).

	Inhibition Zone Diameter (mm)			
	HE1	HE2	CuSO_4_	DMSO
*M. aeruginosa*	12.56 ± 0.82 ^a^	13.66 ± 0.28 ^b^	27.6 ± 0.6 ^c^	0 ± 0 ^d^
*C. sorokiniana*	20.7 ± 0.24 ^a^	20.9 ± 0.63 ^a^	40.34 ± 1.36 ^b^	0 ± 0 ^c^

**Table 3:** Anti-cyanobacterial and anti-microalgal activity of HE1 and HE2 extracts in liquid medium. MIC, minimum inhibitory concentration. MMC, minimum microbicidal concentration.

	MIC (µg/mL) *			MMC (µg/mL) **		
	HE1	HE2	CuSO_4_	HE1	HE2	CuSO_4_
*M. aeruginosa*	375	375	4.6875	375	375	4.6875
*C. sorokiniana*	750	375	4.6875	750	375	4.6875

* MIC values represent the lowest concentrations in the serial dilution series where no visible growth was observed. ** MMC values represent the lowest concentrations tested in the serial dilution series that resulted in no cell regrowth.

**Table 4:** Inhibition rates (%) of HE1 and HE2 extracts at the minimum inhibitory concentration on the growth rate of *M. aeruginosa*. Each value represents the mean ± SD of three replicates. Means with different letters are significantly different (*p* < 0.005). SC, solvent control (DMSO); PC, positive control (CuSO_4_) at the minimum inhibitory concentration.

Treatments	Time (Days)				
	0	2	4	6	8
SC	0 ± 0	6.46 ± 0.19 ^a^	16.08 ± 0.10 ^a^	9.40 ± 0.16 ^a^	7.41 ± 0.19 ^a^
PC	0 ± 0	78.40 ± 0.09 ^b^	76.44 ± 0.02 ^b^	95.56 ± 0.01 ^b^	98.99 ± 0.05 ^b^
HE1	0 ± 0	76.93 ± 0.07 ^c^	82.73 ± 0.03 ^c^	95.68 ± 0.01 ^b^	98.95 ± 0.01 ^bc^
HE2	0 ± 0	79.62 ± 0.12 ^d^	88.69 ± 0.04 ^d^	94.16 ± 0.01 ^c^	98.79 ± 0.01 ^c^

**Table 5:** Inhibition rates (%) of HE1 and HE2 extracts at the minimum inhibitory concentration on the growth rate of *C. sorokiniana*. Each value represents the mean ± SD of three replicates. Means with different letters are significantly different (*p* < 0.005). SC, solvent control (DMSO); PC, positive control (CuSO_4_) at the minimum inhibitory concentration.

Treatments	Time (Days)				
	0	2	4	6	8
SC	0 ± 0	28.99 ± 0.21 ^a^	11.83 ± 0.21 ^a^	14.07 ± 0.17 ^a^	10.61 ± 0.23 ^a^
PC	0 ± 0	81.07 ± 0.09 ^b^	94.91 ± 0.03 ^b^	98.86 ± 0.01 ^b^	98.40 ± 0.01 ^b^
HE1	0 ± 0	88.59 ± 0.04 ^c^	92.52 ± 0.04 ^c^	97.75 ± 0.01 ^c^	98.09 ± 0.01 ^c^
HE2	0 ± 0	90.21 ± 0.10 ^d^	94.10 ± 0.06 ^d^	98.77 ± 0.01 ^b^	98.64 ± 0.01 ^d^

**Table 6:** Binding affinities (kcal/mol) of selected compounds with protein targets.

Compound	Aspartate Racemase (5WXX)	Phycocyanin (1HA7)	AerF Protein (6JH7)	McyG Adenylation–Peptidyl Carrier Protein Didomain (4R0M)	Enoyl-[acyl-carrier-protein] Reductase (1C14)	RuBisCO (8Q04)
Trans-verbenol	−5.2	−6.6	−5.4	−5.4	−5.5	−5.8
Naphthalene	−5.2	−6.3	−6.4	−5.8	−5.5	−5.7
ρ-Cymene-8-ol	−5.8	−6.2	−6.4	−6	−5.8	−5.7
Quinoline	−5.2	−6	−6.2	−5.5	−5.4	−5.7
2-Furancarboxaldehyde	−4.7	−4.3	−4.8	−4.3	−5.3	−4.3
Chloroatranol	−5.1	−5.8	−6.3	−4.7	−5.5	−5.8
δ-Cadinene	−6.1	−7.5	−6.5	−5.4	−6.5	−6.6
Himachalol	−6.3	−7.4	−6.2	−6.1	−6.1	−6.9
Methyl hematommate	−5.2	−6.2	−6.5	−5.4	−6.1	−6.2
Atraric acid	−5.2	−6.3	−7.2	−6.6	−6.1	−6.4
Acetisoeugenol	−5.1	−6.5	−6	−6	−6.7	−6.2
Guaiol acetate	−6.6	−7.1	−6.6	−6.3	−7.1	−7.5
n-Hexadecanoic acid	−4.1	−3.9	−5	−3.9	−5.5	−5.1
Abietatriene	−8.3	−8.3	−7.5	−6.8	−7.5	−8.4
9,12,15-Octadecatrienoic acid	−4.7	−5.6	−5.3	−4.9	−5.8	−5.8

**Table 7:** Summary of ADMET properties of volatile compounds.

	Trans-Verbenol	Naphthalene	ρ-Cymene-8-ol	Quinoline	2-Furancarboxaldehyde	Chloroatranol	δ-Cadinene	Himachalol	Methyl Hematommate	Atraric Acid	Acetisoeugenol	Guaiol Acetate	n-Hexadecanoic Acid	Abietatriene	9,12,15- Octadecatrienoic Acid
Molecular weight	152.23	128.17	150.22	129.16	96.08	186.59	204.35	222.37	210.18	196.2	206.24	264.4	256.42	270.45	278.43
Number of heavy atoms	11	10	10	10	7	12	15	16	15	14	15	19	18	20	20
Number of aromatic heavy atoms	0	10	6	10	5	6	0	0	6	6	6	0	0	6	0
Fraction *Csp*_3_	0.80	0.00	0.40	0	0	0.12	0.73	0.87	0.20	0.30	0.25	0.82	0.94	0.70	0.61
Number of rotatable bonds	0	0	1	0	1	1	1	0	3	2	4	3	14	1	13
Number of H-bond acceptors	1	0	1	1	2	3	0	1	5	4	3	2	2	0	2
Number of H-bond donors	1	0	1	0	0	2	0	1	2	2	0	0	1	0	1
Molar Refractivity	46.38	43.95	47.03	41.74	24.10	45.85	69.04	70.46	52.12	51.70	59.33	80.45	80.80	89.61	88.99
TPSA (Å^2^)	20.23	0.00	20.23	12.89	30.21	57.53	0.00	20.23	83.83	66.76	35.53	26.30	37.30	0.00	37.30
Consensus Log Po/w	2.31	3.1	2.25	2.08	0.69	1.76	4.14	3.49	1.26	1.77	2.71	3.89	5.20	5.88	5.88
Lipinski’s rules	Yes	Yes	Yes	Yes	Yes	Yes	Yes	Yes	Yes	Yes	Yes	Yes	Yes	Yes	Yes
Lipinski’s violation	0 Violations	1 Violation: MLOGP > 4.15	0 Violations	0 Violations	0 Violations	0 Violations	1 Violation: MLOGP > 4.15	0 Violations	0 Violations	0 Violations	0 Violations	0 Violations	1 Violation: MLOGP > 4.15	1 Violation: MLOGP > 4.15	1 Violation: MLOGP > 4.15
Bioavailability Score	0.55	0.55	0.55	0.55	0.55	0.55	0.55	0.55	0.55	0.55	0.55	0.55	0.85	0.55	0.55
GI absorption	High	Low	High	High	High	High	Low	High	High	High	High	High	High	Low	Low
BBB permeant	Yes	Yes	Yes	Yes	Yes	Yes	No	Yes	No	Yes	Yes	Yes	Yes	No	No
P-gp substrate	No	No	No	No	No	No	No	No	No	No	No	No	No	Yes	Yes
CYP1A2 inhibitor	No	Yes	Yes	Yes	No	No	No	No	No	No	Yes	No	Yes	No	No
CYP2C19 inhibitor	No	No	No	No	No	No	Yes	No	No	No	Yes	Yes	No	Yes	Yes
CYP2C9 inhibitor	No	No	No	No	No	No	Yes	No	No	No	No	Yes	Yes	Yes	Yes
CYP2D6 inhibitor	No	No	No	No	No	No	No	No	No	No	No	No	No	No	No
CYP3A4 inhibitor	No	No	No	No	No	Yes	No	No	No	No	No	No	No	No	No
*Log K_p_* (cm/s)	−4.99	−4.74	−5.8	−5.65	−6.6	−5.86	−4.85	−5.18	−6.17	−5.84	−5.3	−5.33	−2.77	−2.93	−2.93
PAINS	0 Alerts	0 Alerts	0 Alerts	0 Alerts	0 Alerts	0 Alerts	0 Alerts	0 Alerts	0 Alerts	0 Alerts	0 Alerts	0 Alerts	0 Alerts	0 Alerts	0 Alerts
Brenk	1 Alert: Isolated Alkene	0 Alerts	0 Alerts	0 Alerts	1 Alert: Aldehyde	1 Alert: Aldehyde	1 Alert: Isolated Alkene	1 Alert: Isolated Alkene	1 Alert: Aldehyde	0 Alerts	1 Alert: Phenol Ester	1 Alert: Isolated Alkene	0 Alerts	0 Alerts	0 Alerts
Synthetic accessibility	4.47	1.00	1.00	1.00	1.77	1.37	4.14	4.14	1.84	1.81	2.11	4.30	2.31	3.18	3.18
Egan’s rule	Yes	Yes	Yes	Yes	Yes	Yes	Yes	Yes	Yes	Yes	Yes	Yes	Yes	Yes	Yes
Veber’s rule	Yes	Yes	Yes	Yes	Yes	Yes	Yes	Yes	Yes	Yes	Yes	Yes	No; 1 Violation: Number of Rotatable Bonds > 10	Yes	Yes

**Table 8:** ProTox-III toxicity predictions (LD_50_ values, toxicity class, organ/toxicological endpoints).

	Hepatotoxicity		Carcinogenicity		Immunotoxicity		Mutagenicity		Cytotoxicity		Predicted LD_50_ (mg/kg)	Toxicity Class
	Pr	Pb	Pr	Pb	Pr	Pb	Pr	Pb	Pr	Pb		
Trans-verbenol	NH	0.79	NC	0.61	NI	0.90	NM	0.93	NCy	0.77	2340	V
Naphthalene	NH	0.83	C	0.88	NI	0.99	M	0.89	NCy	0.91	316	IV
ρ-Cymene-8-ol	NH	0.86	C	0.61	NI	0.99	NM	0.96	NCy	0.89	1020	IV
Quinoline	NH	0.57	NC	0.78	NI	0.94	M	1.00	NCy	0.94	331	IV
2-Furancarboxaldehyde	NH	0.73	C	0.89	NI	0.99	M	0.77	NCy	0.72	65	III
Chloroatranol	NH	0.55	NC	0.71	NI	0.96	NM	0.86	NCy	0.72	660	IV
δ-Cadinene	NH	0.82	NC	0.75	NI	0.66	NM	0.68	NCy	0.69	4390	V
Himachalol	NH	0.79	NC	0.67	I	0.74	NM	0.79	NCy	0.89	265	III
Methyl hematommate	NH	0.61	NC	0.77	NI	0.92	NM	0.90	NCy	0.89	1900	IV
Atraric acid	NH	0.54	NC	0.71	NI	0.81	NM	0.87	NCy	0.83	1900	IV
Acetisoeugenol	H	0.59	NC	0.58	I	0.78	NM	0.53	NCy	0.84	3450	V
Guaiol acetate	NH	0.54	NC	0.50	NI	0.90	NM	0.89	NCy	0.80	4800	V
n-Hexadecanoic acid	NH	0.52	NC	0.63	NI	0.99	NM	1.00	NCy	0.74	900	IV
Abietatriene	NH	0.88	NC	0.74	NI	0.85	NM	0.76	NCy	0.82	6700	VI
9,12,15-Octadecatrienoic acid	NH	0.54	NC	0.63	NI	0.99	NM	0.95	NCy	0.71	10,000	VI

Pr: Prediction, Pb: Probability, NH: Non-hepatotoxic, NC: Non-carcinogenic, NCy: Non-cytotoxic, NI: Non-immunotoxic, NM: Non-mutagenic, H: Hepatotoxic, I: Immunotoxic, C: Carcinogenic. Toxicity class: Class I, fatal if swallowed (LD_50_  ≤  5); Class II, fatal if swallowed (5  <  LD_50_  ≤  50); Class III, toxic if swallowed (50  <  LD_50_  ≤  300); Class IV, harmful if swallowed (300  <  LD_50_  ≤  2000); Class V, may be harmful if swallowed (2000  <  LD_50_  ≤  5000); Class VI, non-toxic (LD_50_  >  5000).

## Data Availability

The original contributions presented in this study are included in the article.

## Abbreviations

The following abbreviations are used in this manuscript:

ADME	Absorption, Distribution, Metabolism, and Excretion
ADMET	Absorption, Distribution, Metabolism, Excretion, and Toxicity
ANOVA	Analysis of Variance
BSA	Bovine Serum Albumin
C	Carcinogenic
CAT	Catalase
CYP	Cytochrome P450
DMSO	Dimethylsulfoxide
H	Hepatotoxic
HABs	Harmful Algal Blooms
HCBs	Harmful Cyanobacterial Blooms
HE1	Volatile Fraction from Pseudevernia furfuracea (High Atlas)
HE2	Volatile Fraction from Pseudevernia furfuracea (Middle Atlas)
HSD	Honest Significant Difference
I	Immunotoxic
IR	Inhibition Rate
IZD	Inhibition Zone Diameter
LD_50_	Median Lethal Dose
MDA	Malondialdehyde
MIC	Minimum Inhibitory Concentration
MMC	Minimum Microbicidal Concentration
NBT	Nitro Blue Tetrazolium
NC	Negative Control/Noncarcinogenic
NCy	Noncytotoxic
NH	Nonhepatotoxic
NI	Nonimmunotoxic
NM	Nonmutagenic
PAINS	Potential Assay Interference and Structural Liabilities
Pb	Probability
PBS	Phosphate Buffer Saline
PC	Positive Control
P-gp	P-glycoprotein
Pr	Prediction
PVP	Polyvinylpyrrolidone
ROS	Reactive Oxygen Species
SC	Solvent Control
SD	Standard Deviation
SOD	Superoxide Dismutase
SPSS	Statistical Package for the Social Sciences
TBA	Thiobarbituric Acid
VOCs	Volatile Organic Compounds

## References

[1] Brenckman C.M., Parameswarappa Jayalakshmamma M., Pennock W.H., Ashraf F., Borgaonkar A.D. (2025). A Review of Harmful Algal Blooms: Causes, Effects, Monitoring, and Prevention Methods. Water.

[2] Huisman J., Codd G.A., Paerl H.W., Ibelings B.W., Verspagen J.M.H., Visser P.M. (2018). Cyanobacterial blooms. Nat. Rev. Microbiol..

[3] Igwaran A., Kayode A.J., Moloantoa K.M., Khetsha Z.P., Unuofin J.O. (2024). Cyanobacteria Harmful Algae Blooms: Causes, Impacts, and Risk Management. Water Air Soil Pollut..

[4] Wang Y., Zhao D., Woolway R.I., Yan H., Paerl H.W., Zheng Y., Zheng C., Feng L. (2025). Global elevation of algal bloom frequency in large lakes over the past two decades. Natl. Sci. Rev..

[5] Amorim C.A., Moura A.D.N. (2021). Ecological impacts of freshwater algal blooms on water quality, plankton biodiversity, structure, and ecosystem functioning. Sci. Total Environ..

[6] Anabtawi H.M., Lee W.H., Al-Anazi A., Mohamed M.M., Aly Hassan A. (2024). Advancements in Biological Strategies for Controlling Harmful Algal Blooms (HABs). Water.

[7] Lürling M., Mucci M. (2020). Mitigating eutrophication nuisance: In-lake measures are becoming inevitable in eutrophic waters in the Netherlands. Hydrobiologia.

[8] Goga M., Elečko J., Marcinčinová M., Ručová D., Bačkorová M., Bačkor M. (2020). Lichen Metabolites: An Overview of Some Secondary Metabolites and Their Biological Potential. Reference Series in Phytochemistry.

[9] Singh G., Dal Grande F., Martin F.M., Medema M.H. (2025). Breaking into nature’s secret medicine cabinet: Lichens—A biochemical goldmine ready for discovery. New Phytol..

[10] Pasinato A., Singh G. (2025). Lichens are a treasure chest of bioactive compounds: Fact or fake?. New Phytol..

[11] Adenubi O.T., Famuyide I.M., McGaw L.J., Eloff J.N. (2022). Lichens: An update on their ethnopharmacological uses and potential as sources of drug leads. J. Ethnopharmacol..

[12] Fernández-Moriano C., Gómez-Serranillos M.P., Crespo A. (2016). Antioxidant potential of lichen species and their secondary metabolites. A systematic review. Pharm. Biol..

[13] Furmanek L., Seaward M.R.D. (2023). Anti-yeast potential of lichen-extracted substances- An analytical review. S. Afr. J. Bot..

[14] Honegger R. (1991). Functional aspects of the lichen symbiosis. Annu. Rev. Plant Physiol. Plant Mol. Biol..

[15] Spribille T., Tuovinen V., Resl P., Vanderpool D., Wolinski H., Aime M.C., Schneider K., Stabentheiner E., Toome-Heller M., Thor G. (2016). Basidiomycete yeasts in the cortex of ascomycete macrolichens. Science.

[16] Pichler G., Muggia L., Carniel F.C., Grube M., Kranner I. (2023). How to build a lichen: From metabolite release to symbiotic interplay. New Phytol..

[17] Ahmadjian V. (1993). The Lichen Symbiosis.

[18] Aschenbrenner I.A., Cernava T., Berg G., Grube M. (2016). Understanding microbial multi-species symbioses. Front. Microbiol..

[19] Bačkor M., Goga M., Ručová D., Urminská D., Bačkorová M., Klejdus B. (2023). Allelopathic effects of three lichen secondary metabolites on cultures of aposymbiotically grown lichen photobionts and free-living alga Scenedesmus quadricauda. S. Afr. J. Bot..

[20] Bačkor M., Klemová K., Bačkorová M., Ivanova V. (2010). Comparison of the Phytotoxic Effects of Usnic Acid on Cultures of Free-Living Alga Scenedesmus quadricauda and Aposymbiotically Grown Lichen Photobiont *Trebouxia erici*. J. Chem. Ecol..

[21] Gazzano C., Favero-Longo S.E., Iacomussi P., Piervittori R. (2013). Biocidal effect of lichen secondary metabolites against rock-dwelling microcolonial fungi, cyanobacteria and green algae. Int. Biodeterior. Biodegrad..

[22] Lokajová V., Bačkorová M., Bačkor M. (2014). Allelopathic effects of lichen secondary metabolites and their naturally occurring mixtures on cultures of aposymbiotically grown lichen photobiont *Trebouxia erici* (Chlorophyta). S. Afr. J. Bot..

[23] Legrand C., Rengefors K., Fistarol G.O., Granéli E. (2003). Allelopathy in phytoplankton—Biochemical, ecological and evolutionary aspects. Phycologia.

[24] Essadki Y., Hilmi A., Cascajosa-Lira A., Girão M., Darrag E.M., Martins R., Romane A., El Amrani Zerrifi S., Mugani R., Tazart Z. (2024). In Vitro Antimicrobial Activity of Volatile Compounds from the Lichen *Pseudevernia furfuracea* (L.) Zopf. Against Multidrug-Resistant Bacteria and Fish Pathogens. Microorganisms.

[25] El Amrani Zerrifi S., El Khalloufi F., Mugani R., El Mahdi R., Kasrati A., Soulaimani B., Barros L., Ferreira I.C.F.R., Amaral J.S., Finimundy T.C. (2020). Seaweed Essential Oils as a New Source of Bioactive Compounds for Cyanobacteria Growth Control: Innovative Ecological Biocontrol Approach. Toxins.

[26] Zerrifi S.E.A., Kasrati A., Redouane E.M., Tazart Z., Khalloufi F.E., Abbad A., Oudra B., Campos A., Vasconcelos V. (2020). Essential oils from Moroccan plants as promising ecofriendly tools to control toxic cyanobacteria blooms. Ind. Crops Prod..

[27] Zhang Q., Brambilla E., Li R., Shi H., Cosentino Lagomarsino M., Sclavi B. (2020). A Decrease in Transcription Capacity Limits Growth Rate upon Translation Inhibition. mSystems.

[28] Zerrifi S.E.A., Redouane E.M., Mugani R., Ribeiro I., De Fátima Carvalho M., Campos A., Barakate M., Vasconcelos V., Oudra B., El Khalloufi F. (2021). Moroccan actinobacteria with promising activity against toxic cyanobacteria Microcystis aeruginosa. Environ. Sci. Pollut. Res..

[29] Wu L., Qiu Z., Zhou Y., Du Y., Liu C., Ye J., Hu X. (2016). Physiological effects of the herbicide glyphosate on the cyanobacterium Microcystis aeruginosa. Aquat. Toxicol..

[30] Bačkor M., Kecsey D., Drábová B., Urminská D., Šemeláková M., Goga M. (2024). Secondary Metabolites from Australian Lichens Ramalina celastri and Stereocaulon ramulosum Affect Growth and Metabolism of Photobiont Asterochloris erici through Allelopathy. Molecules.

[31] Lins L., Dal Maso S., Foncoux B., Kamili A., Laurin Y., Genva M., Jijakli M.H., De Clerck C., Fauconnier M.L., Deleu M. (2019). Insights into the Relationships Between Herbicide Activities, Molecular Structure and Membrane Interaction of Cinnamon and Citronella Essential Oils Components. Int. J. Mol. Sci..

[32] Sanad H., Belattmania Z., Nafis A., Hassouani M., Mazoir N., Reani A., Hassani L., Vasconcelos V., Sabour B. (2022). Chemical Composition and In Vitro Antioxidant and Antimicrobial Activities of the Marine Cyanolichen *Lichina pygmaea* Volatile Compounds. Mar. Drugs.

[33] Zerrifi S.E., Tazart Z., El Khalloufi F., Oudra B., Campos A., Vasconcelos V. (2019). Potential control of toxic cyanobacteria blooms with Moroccan seaweed extracts. Environ. Sci. Pollut. Res..

[34] Uchida H., Kouchiwa T., Watanabe K., Kawasaki A., Hodoki Y., Ohtani I.I., Yamamoto Y., Suzuki M., Harada K.-I. (1998). A Coupled Assay System for the Lysis of Cyanobacteria. Jpn. J. Water Treat. Biol..

[35] Sbiyyaa B., Loudiki M., Oudra B. (1998). Capacité de stockage intracellulaire de l’azote et du phosphore chezMicrocystis aeruginosa Kütz. et *Synechocystis* sp.: Cyanobactériestoxiques occasionnant des blooms dans la région de Marrakech (Maroc). Ann. De Limnol.-Int. J. Limnol..

[36] Xu H., Paerl H.W., Qin B., Zhu G., Gaoa G. (2010). Nitrogen and phosphorus inputs control phytoplankton growth in eutrophic Lake Taihu, China. Limnol. Oceanogr..

[37] Yéprémian C., Catherine A., Bernard C., Congestri R., Elersek T., Pilkaityte R. (2016). Chlorophyll a Extraction and Determination. Handbook of Cyanobacterial Monitoring and Cyanotoxin Analysis.

[38] Li J., Liu Y., Zhang P., Zeng G., Cai X., Liu S., Yin Y., Hu X., Hu X., Tan X. (2016). Growth inhibition and oxidative damage of Microcystis aeruginosa induced by crude extract of Sagittaria trifolia tubers. J. Environ. Sci..

[39] Bradford M.M. (1976). A rapid and sensitive method for the quantitation of microgram quantities of protein utilizing the principle of protein-dye binding. Anal. Biochem..

[40] Beauchamp C., Fridovich I. (1971). Superoxide dismutase: Improved assays and an assay applicable to acrylamide gels. Anal. Biochem..

[41] Rao M.V., Paliyath G., Ormrod D.P. (1996). Ultraviolet-B- and Ozone-Induced Biochemical Changes in Antioxidant Enzymes of Arabidopsis thaliana. Plant Physiol..

[42] Hodges D.M., Delong J.M., Forney C.F., Prange R.K. (1999). Improving the thiobarbituric acid-reactive-substances assay for estimating lipid peroxidation in plant tissues containing anthocyanin and other interfering compounds. Planta.

[43] Padyana A.K., Bhat V.B., Madyastha K., Rajashankar K., Ramakumar S. (2001). Crystal structure of a light-harvesting protein C-phycocyanin from Spirulina platensis. Biochem. Biophys. Res. Commun..

[44] Qiu X., Abdel-Meguid S.S., Janson C.A., Court R.I., Smyth M.G., Payne D.J. (1999). Molecular basis for triclosan activity involves a flipping loop in the active site. Protein Sci..

[45] Barrett J., Naduthodi M.I., Mao Y., Dégut C., Musiał S., Salter A., Leake M.C., Plevin M.J., McCormick A.J., Blaza J.N. (2024). A promiscuous mechanism to phase separate eukaryotic carbon fixation in the green lineage. Nat. Plants.

[46] Cao D.-D., Zhang C.-P., Zhou K., Jiang Y.-L., Tan X.-F., Xie J., Ren Y.-M., Chen Y., Zhou C.-Z., Hou W.-T. (2019). Structural insights into the catalysis and substrate specificity of cyanobacterial aspartate racemase McyF. Biochem. Biophys. Res. Commun..

[47] Qiu X., Wei Y., Zhu W., Fu J., Duan X., Jin H., Zhu P., Zhou C., Yan X. (2020). Structural and functional investigation of AerF, a NADPH-dependent alkenal double bond reductase participating in the biosynthesis of Choi moiety of aeruginosin. J. Struct. Biol..

[48] Tan X.-F., Dai Y.-N., Zhou K., Jiang Y.-L., Ren Y.-M., Chen Y., Zhou C.-Z. (2015). Structure of the adenylation–peptidyl carrier protein didomain of the Microcystis aeruginosa microcystin synthetase McyG. Biol. Crystallogr..

[49] Berman H.M. (2000). The Protein Data Bank. Nucleic Acids Res..

[50] Kim S., Thiessen P.A., Bolton E.E., Chen J., Fu G., Gindulyte A., Han L., He J., He S., Shoemaker B.A. (2016). PubChem Substance and Compound databases. Nucleic Acids Res..

[51] Dallakyan S., Olson A.J. (2015). Small-Molecule Library Screening by Docking with PyRx.

[52] Daina A., Michielin O., Zoete V. (2017). SwissADME: A free web tool to evaluate pharmacokinetics, drug-likeness and medicinal chemistry friendliness of small molecules. Sci. Rep..

[53] Egan W.J., Merz K.M., Baldwin J.J. (2000). Prediction of Drug Absorption Using Multivariate Statistics. J. Med. Chem..

[54] Veber D.F., Johnson S.R., Cheng H.-Y., Smith B.R., Ward K.W., Kopple K.D. (2002). Molecular Properties That Influence the Oral Bioavailability of Drug Candidates. J. Med. Chem..

[55] Lipinski C.A. (2004). Lead- and drug-like compounds: The rule-of-five revolution. Drug Discov. Today Technol..

[56] Baell J.B., Holloway G.A. (2010). New Substructure Filters for Removal of Pan Assay Interference Compounds (PAINS) from Screening Libraries and for Their Exclusion in Bioassays. J. Med. Chem..

[57] Brenk R., Schipani A., James D., Krasowski A., Gilbert I.H., Frearson J., Wyatt P.G. (2008). Lessons Learnt from Assembling Screening Libraries for Drug Discovery for Neglected Diseases. ChemMedChem.

[58] Banerjee P., Kemmler E., Dunkel M., Preissner R. (2024). ProTox 3.0: A webserver for the prediction of toxicity of chemicals. Nucleic Acids Res..

